# Predictive Modeling of Global SARS-CoV-2 Infection Risk in Animals: Unveiling Potential Reservoirs and Informing Health Policy Synergies

**DOI:** 10.1155/tbed/3959370

**Published:** 2025-09-30

**Authors:** Ruying Fang, Luqi Wang, Xin Yang, Yiyang Guo, Bingjie Peng, Yinsheng Zhang, Dilinuer Kamili, Sirui Li, Yunting Lyv, Sen Li, Shunqing Xu

**Affiliations:** ^1^Institute of Artificial Intelligence, Huazhong University of Science and Technology, Wuhan, China; ^2^School of Environmental Science and Engineering, Huazhong University of Science and Technology, Wuhan, China; ^3^Department of Environment Science and Engineering, Fudan University, Shanghai, China; ^4^Key Laboratory of Environment and Health, Ministry of Education, School of Public Health, Tongji Medical College, Huazhong University of Science and Technology, Wuhan, China

**Keywords:** animal infection, distribution, machine learning, mapping, SARS-CoV-2

## Abstract

Reports of SARS-CoV-2 infections in animals have increasingly raised concerns about potential natural reservoirs for the virus. However, our understanding of the global distribution and drivers of animal infection risk remains limited. To bridge this knowledge gap, we conducted extensive data mining from various sources and developed machine learning (ML) models to estimate the global probability of SARS-CoV-2 infections in animals. We trained and evaluated three ML models, mapping the distribution of infection risk in well-documented regions and projecting risk in areas with sparse infection records. Our models pinpointed high-risk areas in Europe and the United States, where infection records are scattered, as well as in the southern regions of Brazil and Asia, which have sparse infection records. Notably, our projections indicated overlaps between predicted high-risk areas and the known distribution of white-tailed deer, American minks, and Asian small-clawed otters. Anthropogenic factors were found to be more predictive of animal infection than biophysical factors, highlighting the importance of accessibility, population density, and COVID-19 mortality rates. These findings suggest the potential for synergies between public and animal health policies.

## 1. Introduction

Despite no longer being classified as a global health emergency, the COVID-19 pandemic continues to pose a significant threat to both public and animal health. The dynamic nature of the severe acute respiratory syndrome coronavirus 2 (SARS-CoV-2) virus, along with its persistent fatality risk, underscores the urgent need to strengthen surveillance and control strategies to mitigate the risk of natural reservoir formation. It is crucial to intensify efforts across various disciplines to effectively contain and minimize the global health impact of COVID-19.

Accumulating evidence strongly suggests that SARS-CoV-2 is a zoonotic disease capable of interspecies transmission [1]. Initially, theories posited two potential pathways for the emergence of SARS-CoV-2; natural selection within a human following zoonotic acquisition or natural selection within an intermediate animal host preceding zoonotic transmission to humans [2]. Human lives are at risk, and there is a potential threat to the lives of animals as well. The overall mortality rate of animals infected with SARS-CoV-2, typically less than 2%, increases significantly in instances of large-scale deaths, particularly on mink farms due to capture and culling practices. There might be a risk for conservation efforts when animals with high conservation value die (e.g., in zoos). The infections in animals are indeed linked to human infections, as most animal infections result from human-to-animal transmission. However, it is important to mention the proximity of humans and animals, which is influenced by human behavior and cultural context, as a significant risk factor. SARS-CoV-2 has been detected in domestic and stray cats [3], lions, and tigers [4], domestic ferrets on fur farms [5], also in American mink [6] and white-tailed deer [7] in the wild. Subsequent studies have confirmed widespread SARS-CoV-2 RNA detection in free-ranging white-tailed deer populations, with infection rates reaching 33%–36% in multiple U.S. states [8, 9]. Recent examples of the transmission of this virus from infected humans to animals have raised concerns about the potential spread of the virus from humans to animals. So far, transmission has been documented in the golden hamster, cat, white-tailed deer, and mink. Additionally, the reverse transmission possibility from companion animals to humans can significantly contribute to the widespread distribution of COVID-19 infection [10, 11]. The most significant example of SARS-CoV-2 spillover and spillback events occurred in mink farming operations, particularly in Denmark, the Netherlands, and other European countries. SARS-CoV-2 transmission from humans to farmed mink was followed by rapid spread within mink populations and subsequent spillback to humans, with evidence of virus mutations that raised concerns about vaccine efficacy [12–14]. These events led to the culling of over 17 million mink in Denmark alone and millions more across affected European countries, representing one of the largest animal culling operations in response to a zoonotic disease threat [15]. The mink-associated SARS-CoV-2 variants demonstrated the potential for the virus to evolve in animal hosts and subsequently reenter human populations, highlighting the critical importance of monitoring animal reservoirs [16]. Only one such event has been reported in hamster [10], the risk is considered to be very low.

Gaining an understanding of the environmental factors that drive the emergence, zoonotic spillover, and spillback of SARS-CoV-2 is crucial for guiding future prevention and control strategies against similar pathogens. Several biophysical and anthropogenic factors are believed to influence the ecoepidemiology of the SARS-CoV-2 virus. Biophysical factors encompass landscape features and environmental conditions, such as landscape characteristics [17], land use [18, 19], temperature [20, 21], humidity [22], precipitation [23], solar irradiance [24], and wind speed [25]. Anthropogenic factors include elements such as direct contact [26], the presence of wildlife markets [27], sociodemographic traits, medical conditions, and economic context [28]. Illegal trade of wildlife is also an important risk factor. Data availability is very limited on this. Furthermore, it is crucial to deploy advanced epidemiological models for forecasting the future geographic hotspots of the disease in the human population [29]. However, limited effort has been made in modeling animal SARS-CoV-2 infection. To accurately predict the transmission dynamics of the virus in animals sharing habitats with humans, a comprehensive understanding of each contributing factor is essential. These measures will provide valuable insights for public health interventions and risk management strategies, aiming to mitigate the likelihood of future spillover events.

To establish a connection between animal SARS-CoV-2 cases and environmental risk factors and to identify the current and potential distribution in locations with limited data, a predictive modeling study was essential for improving disease response, prevention at source and emergency preparedness. Employing machine learning (ML) techniques, specifically Boosted Regression Tree (BRT), eXtreme Gradient Boosting (XGBoost), and Random Forests (RFs), we investigated the biophysical and anthropogenic factors influencing SARS-CoV-2 infection in targeted (IUCN) animal species. Variables include population density, COVID-19 mortality, urban accessibility (travel time to cities), ecoclimatic conditions, livestock density, and land-use patterns. We assessed the accuracy of our models in predicting the global distribution of animal SARS-CoV-2 infections and visualized the projected risk. Assessing susceptible species' geographic distribution and overlap with higher infection risk highlights potential pathogen reservoirs in wild animals, acting as potential sources for human and domestic animal infection.

## 2. Methods

### 2.1. Compilation of a Georeferenced Dataset of SARS-CoV-2 Infections in Animals

A comprehensive literature search for animal SARS-CoV-2-related studies was conducted using major databases such as the Web of Science core database, Embase, and PubMed, covering works up to December 2022 (Figure S1). The search terms used were (“SARS-CoV-2” [Title/Keywords/Abstract/]) AND (“animal” [Title/Keywords/Abstract/]). Two authors reviewed the abstracts for screening, and any disagreement on inclusion was reconciled through consensus. The search also included reports on SARS-CoV-2 in animals from the World Organisation for Animal Health (WOAH) and supplemented the search with results from the GenBank Nucleotide database. Previous research teams have utilized machine extraction models to produce databases, which offer the advantage of rapid data processing and scalability but may suffer from accuracy issues and an inability to capture context-specific information; however, this study opted for collecting information from literature releases, manually extracting, and screening it to ensure rigor. While manual extraction is more time-consuming and labor-intensive, it provides higher accuracy, better quality control, and the ability to capture nuanced information that automated methods might miss. The dataset collected includes information on the animal species, location, and date of diagnosis, and studies focused on clinical trials of SARS-CoV-2 treatment drugs or viral genetic sequencing analysis were excluded. We only collect the data of natural infection. In total, 236 animal SARS-CoV-2 infection records were compiled from 239 papers (literature search covering works up to December 2022), while 388 infection records were gathered from 15 reports published by WHO (reports available up to March 2023) and 57 infection records were retained from a total of 625 studies in the GenBank database (accessed up to March 2023). After removing duplicate cases, the data collected from the three sources were aggregated to summarize a total of 550 records. Each record refers to a single report of the same species in the same place at the same time. In cases where specific coordinates were provided in the text, the locations were labeled accordingly. For locations where no geographic coordinates were provided, the administrative coordinates of the region as expressed in the text were utilized. The georeferencing approach used previously [30] was employed to analyze these cases and identify the initial manifestation locations.

### 2.2. Identification and Analysis of Biophysical and Anthropogenic Factors

In our previous study, factors reported to be associated with SARS-CoV-2 infection were gathered through literature review for further data collection and analysis in this study [31]. We classified all the biophysical and anthropogenic influencing factors into six categories, which are climatic condition, landscape characteristics, livestock density, population density, biodiversity, susceptible animal distribution, COVID-19 mortality, and urban accessibility (travel time to cities). They comprise a total of 27 variables that could potentially affect the SARS-CoV-2 infections in animals and/or have links with the survival and transmission of the COVID-19 virus in humans (Table 1). Driving factors were situated and rescaled to a 0.25° cellular grid.

Biophysical factors, encompassing climate and landscape features, significantly impact the ecological transmission of the SARS-CoV-2 virus and determine the distribution of wildlife species vulnerable to infection. To better understand these factors, we gathered bioclimatic variables (10') from the World Climate Database (WorldClim), which were modeled using meteorological records from 2020. Furthermore, to explore the overlap between habitats of susceptible wild animals and areas with a high risk of SARS-CoV-2 transmission, susceptible animals were identified from literature cited in the previous study [31], and their habitats were further collected and analyzed in this study. We utilized data from the IUCN Red List of threatened species to collect nine animal categories which are susceptible to SARS-CoV-2, including American mink (*Neovison vison*), black-footed ferret (*Mustela nigripes*), western gorilla (*Gorilla gorilla*), lion (*Panthera leo*), snow leopard (*Panthera uncia*), tiger (*Panthera tigris*), white-tailed deer (*Odocoileus virginianus*), Asian small-clawed otter (*Aonyx cinereus*), and puma (*Puma concolor*). Additionally, we incorporated a previously published dataset on global biodiversity intactness, which considered the overall abundance of organisms and their compositional similarity [33].

Anthropogenic factors play a pivotal role in shaping the patterns of human–animal contact across different scales. To enhance our understanding of these factors, we obtained data on human population density from the Socioeconomic Data and Applications Center (SEDAC) using the Gridded Population of the World, Version 4 (GPWv4) for the year 2020. We also gathered landscape characteristics data from the Copernicus Global Land Service, which provided information on common forest types. The landscape characteristics data were assessed at a resolution of 100 m to obtain information within a grid size of 0.25° × 0.25°. Livestock density data were obtained from published articles, while COVID-19 mortality rates and accessibility information were sourced from publicly available databases [32, 34, 35].

### 2.3. Development of Predictive ML Models for Animal Infection

The set of 27 variables was employed in three distinct ML models to assess their predictive capacity for identifying animal infections. Our analysis aimed to gauge and compare the accuracy and efficiency of these ML models, which have previously demonstrated effectiveness in predicting disease outbreaks on a macroscale within the field of spatial epidemiology [36–38].

The BRT model employs a binary outcome as the dependent variable and utilizes environmental variables as independent or predictor variables. This model, widely used in ecological niche modeling [39, 40], demonstrates high effectiveness in predicting organism distributions, while considering nonlinear relationships and interactions among covariates [41, 42]. The XGBoost method exhibits broad applicability across various natural science and biomedical domains due to its powerful computational capabilities and the ability to optimize model performance through parameter tuning [43, 44]. Meanwhile, the RFs model, a decision tree-based method that works by creating numerous trees and combining their outputs to form forests, is an effective solution to the issue of overfitting [45]. RF is capable of handling large datasets and provides a measure of importance that to some extent, applies to causal inference. In our study, we implemented self-referencing functions for both XGBoost and RF models, allowing for autonomous optimization of model parameters.

**Figure 1:**
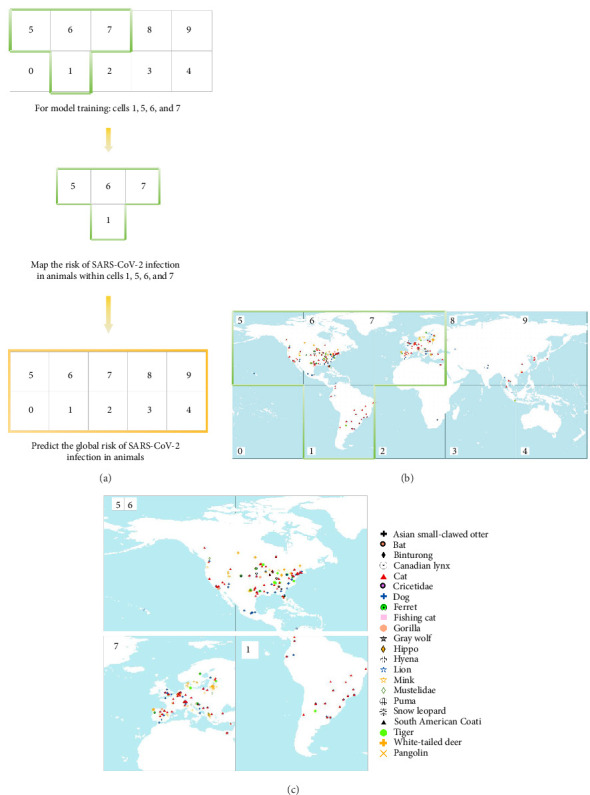
Global distribution of reported SARS-CoV-2 infections in animal (A), and regions utilized for training and testing machine learning models (B), focusing on areas with higher case reporting (C).

Due to data gaps in Australia, African countries, and Asia, which are related to limited resource availability or underreporting to WOAH or via scientific publications, we utilized data-rich regions for model training. We utilized data-rich regions, such as zones 1, 5, 6, and 7, to train the model and predict the risk profile for those regions. Subsequently, we extended the model for risk mapping of global regions (Figure 1A).

Our aim was to then extrapolate these predictions to the global scale. Following an approach adopted by previous studies [36, 46], we generated pseudo-absences to train our models. These pseudo-absences were randomly selected and included in the models if they met two criteria: (i) the cells did not report any animal SARS-CoV-2 occurrences and (ii) they were located at least two decimal degrees [46] away from any positive cells.

To address potential biases introduced by low (<10%) and high (>90%) frequency distributions of binary outcome categories, we applied bootstrapping methods [47, 48]. We conducted a total of 50 iterations to obtain more robust estimates for our performance measures. The iterations involved: (i) creating a balanced sub-sample of data from the entire dataset by including all *n* points with animal SARS-CoV-2 presence and randomly selecting an equal number of points with absence from the “absence” points; (ii) dividing the balanced subset into two sets: a training set comprising 75% of the points and a test set with the remaining 25%; (iii) developing models using the training set with the BRT, XGBoost, and RF methods, while saving their respective parameters; (iv) evaluating the models by using ROC curves and calculating the areas under the curve based on predictions made from the test set and model equations; (v) generating risk maps by saving the average predictions of each model as maps for each of the 50 iterations. Upon completing steps (i) to (v) for 50 iterations, we calculated the mean, standard deviation, and expected spatial distributions.

## 3. Results

### 3.1. Global Patterns of Reported Animal Infections

A total of 550 confirmed outbreak reports of SARS-CoV-2 infections in animals (22 animal species and 26,970 cases) have been documented since its discovery in December 2019 (Figure 1B). Primarily, animal infections have been reported in North America, South America, and Europe. Among the countries that have identified animal infections, seven nations, namely Greece, the Netherlands, the United States of America, Lithuania, Canada, Spain, and Poland, have recorded over 100 cases (Figure 1C). In the United States, 569 cases of SARS-CoV-2 infection were identified across 18 different animal species, with the highest number of cases among all countries examined (Table S1).

### 3.2. Performance of ML Models

**Figure 2:**
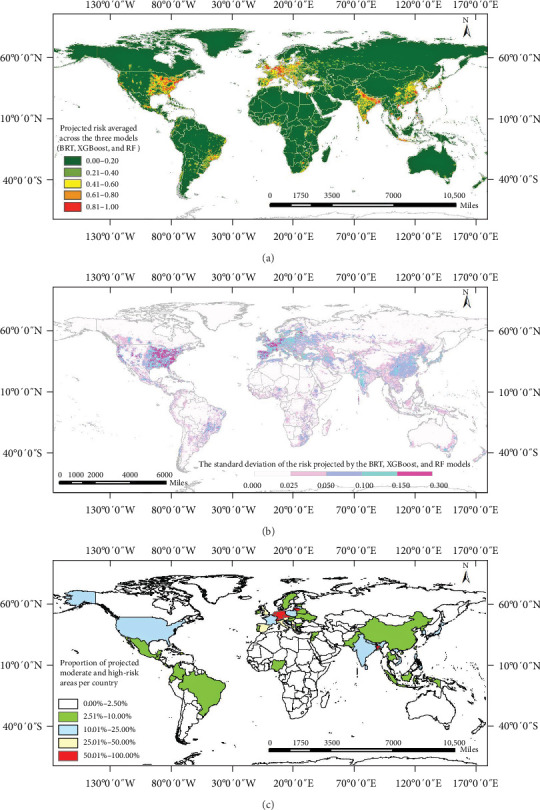
The projected global risk of SARS-CoV-2 infection in animals. (A) Average projected risk of animal infections. (B) Standard deviation of the infection risk distribution across the three models. (C) Percentage of predicted moderate and high-risk areas for animal infections by country.

**Figure 3:**
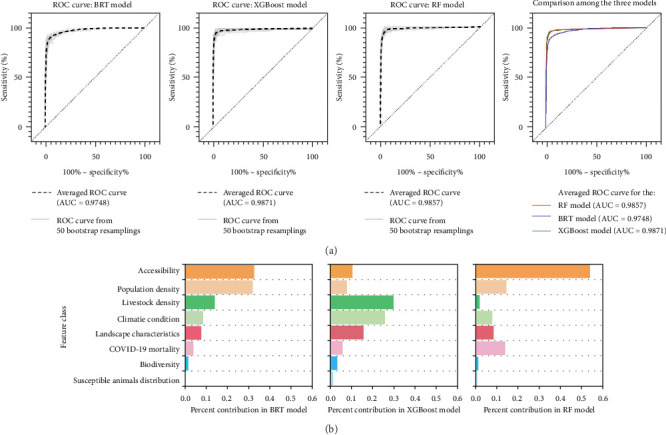
Predictive performance and key drivers of change in machine learning models. (A) ROC curves depicting predicted risk of SARS-CoV-2 infection in animals by machine learning models. (B) Relative importance of key drivers of change in influencing predictive power.

A comparison of the results from all models was conducted using mean and standard deviation plots (Figure 2). Europe and the southern region of North America exhibited higher differences through three models, with the standard deviation reaching a maximum value of 0.27. Coastal regions displayed greater variance compared to inland regions in all three models (Figure 2B). The AUC values for the three models ranged from 0.9659 to 0.9970, indicating good to excellent accuracy for the models' predictions on risk area. The BRT model had a mean AUC value of 0.9748 (Figure 3A), while the XGBoost and RF models performed even better with AUC values of 0.9871 and 0.9857, respectively. These results demonstrate that the XGBoost model outperforms the other ML techniques in predicting the probability of disease occurrence.

Our ML models consistently indicated that the USA and India had the largest high-risk areas, while China ranked third in the BRT prediction model, and Germany ranked third in the XGBoost and RF models (Table S2, Figure 2C). Notably, differences in the size range and the percentage of high-risk areas predicted by the three models highlight the variations in preparedness and capacity among different countries (Figure S2). Specifically, the BRT model predicted narrower ranges of high-risk areas than the other two models. These differences were more pronounced in America and Europe, where Germany's high-risk area accounted for 8.64% of its country's area in the BRT model, compared to 41.87% and 36.60% in the XGBoost and RF models, respectively (Figure S2, Table S2). We found that high-risk areas in Belgium, Bangladesh, Germany, and Switzerland were more extensive, with Belgium accounting for half of the country's high-risk area, emphasizing the need to prioritize animal health issues in those areas (Table S3, Figure 2C).

### 3.3. Projected Risk Distribution of Animal Infections

The average risk map (Figure 2A) revealed that Europe (particularly Western and Central Europe), the Eastern United States, Southern Brazil, and Southern Asia were all high-risk areas for SARS-CoV-2 infections in animals. Within Europe, the United Kingdom, Belgium, the Netherlands, and Germany had multiple concentrated high-risk regions. In Asia, the coastal regions of China, northern India, and Japan displayed high-risk areas. Northern Asia's Russian region had a few scattered medium-risk areas but no high-risk regions.

Analyzing the percentage of high-risk areas in relation to the overall size of the country reveals that Belgium had the highest proportion of high-risk areas (55.42%), followed by the Netherlands, Germany, and Switzerland, with over 20% of their land areas designated as high-risk areas. Additionally, the United States, India, and China had a large number of high-risk areas, although the percentage was lower (<10%) due to their larger geographical areas (Table S3). Figure 2C illustrates the percentage of predicted high- and moderate-risk areas of SARS-CoV-2 infection in animals by country, with a percentage greater than 50% shown in red. Among the countries in red were the Netherlands, Belgium, Poland, Switzerland, and Italy.

The substantial proportion of high-risk areas within Belgium, Bangladesh, Germany, and Switzerland raises concerns about the well-being of local animal populations. Several factors may explain the particularly high-risk projections for these European countries. First, these nations are characterized by exceptionally high population densities and extensive transportation networks, resulting in high accessibility scores—the primary driver identified in our models. Belgium and the Netherlands, in particular, have among the highest population densities in Europe, facilitating frequent human–animal interactions. Second, these countries experienced significant COVID-19 mortality rates during the pandemic, which our models identified as a key predictor of animal infection risk, likely reflecting the intensity of viral circulation and opportunities for spillover events. Third, the geographic compactness of these nations means that high-risk areas represent a larger proportion of their total territory compared to larger countries with similar absolute numbers of high-risk cells. Fourth, these countries have well-developed veterinary surveillance systems and reporting mechanisms, potentially leading to better detection and documentation of animal infections, which may have influenced our training data. Finally, intensive livestock farming practices, particularly mink farming in the Netherlands and Denmark, and the close proximity of agricultural areas to urban centers may have created favorable conditions for human-to-animal transmission events that informed our predictive models.

### 3.4. Factors as Key Drivers of Change

The findings revealed a robust correlation between population density, accessibility, COVID-19 mortality, livestock farming density, and landscape characteristics, the occurrence of SARS-CoV-2 infection among animals (Figure 3B). The three models showed different patterns in factor importance: while accessibility and population density consistently ranked highest across all models, climatic conditions (including precipitation, solar radiation, and wind speed) demonstrated relatively higher importance in the XGBoost model compared to the BRT and RF models, with precipitation showing weights of 5% in XGBoost versus 2% in both BRT and RF (Table S4). Notably, our findings demonstrate that anthropogenic factors exhibit greater predictive capability for animal infection than the biophysical factors examined in our study. Among these factors, accessibility (travel time to cities) ranked the highest, accounting for 32.53% in BRT, 10.58% in XGBoost, and 53.35% in RF. Regions with high accessibility exhibited increased population mobility, thereby elevating the likelihood of animals coming into contact with the virus. Population density consistently ranked as the second highest factor across all models, with weights of 31.83%, 7.89%, and 14.30% (Table S4). COVID-19 mortality emerged as the third highest weighted variable, signifying a direct transmission pathway to animals via human contact in areas characterized by high population density and accessibility. These factors reflect the influence of economic and medical development, advanced research, and nucleic acid testing services, which facilitate the detection of animal infections. Table S4 presents a summary of the highest, lowest, and average weight values across the 50 iterations for each influencing factor.

### 3.5. Susceptible Animal Species in Projected Risk Areas

**Figure 4:**
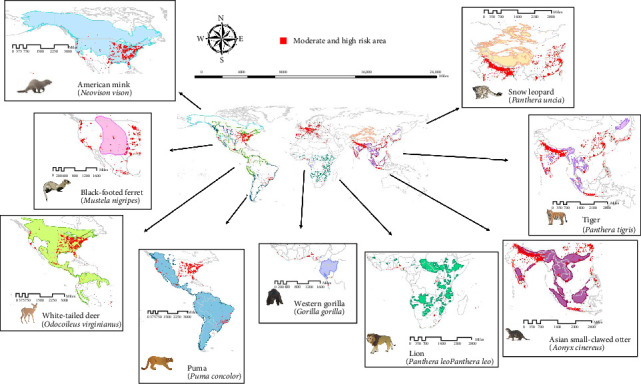
Overlap between distribution of susceptible animal species and projected areas of moderate to high risk for SARS-CoV-2 infection.

We collated data on the biogeographical distribution of nine animal species known to be susceptible to SARS-CoV-2 and mapped their overlap with moderate- and high-risk areas, as shown in Figure S3.  Figure 4 identifies the particular animal species at high risk of SARS-CoV-2 infection, aiming to assist countries with high-risk areas in strengthening their measures for animal protection. The southeastern portion of the American mink's habitat, spanning Canada and the United States, exhibits a significant number of high-risk zones. The habitat of the white-tailed deer extensively overlaps with that of the American mink, encompassing threatened areas in Canada, the United States, as well as Brazil, Colombia, Guatemala, and other regions. Similarly, the fragmented habitat of the Asian small-clawed otter presents numerous high-risk areas across countries such as Bangladesh, China, India, Indonesia, Malaysia, Nepal, Thailand, and Vietnam (Table S5). Although the western gorilla, lion, and snow leopard have only a few high-risk areas within their habitats, they are in proximity to some of these high-risk regions.

## 4. Discussion

Confirmed cases of animal SARS-CoV-2 infections are primarily concentrated in the Americas and Europe, with a limited number of reported cases. Surveillance efforts primarily focus on domestic pets (cats, dogs, and hamsters) and farm animals (mink and ferrets), while reports of infections in large animals mainly come from zoos. Platforms such as the WOAH and Pubpro provide aggregated and self-reported data. Only a limited number of records were obtained from institutions that regularly conduct testing [49]. However, the existing dataset's ability to capture the underdetection of SARS-CoV-2 infection in animals worldwide remains an unresolved issue due to a lack of empirical evidence. This underscores the importance of predictive investigations in shedding light on areas with limited data, especially by leveraging recent advancements in ML-based approaches.

The analysis revealed that the accessibility was the primary determinant of SARS-CoV-2 risk in animals across all models, with BRT having the highest weight (32.53%) and XGBoost and RF having average weights of 10.58% and 53.35%, respectively (Figure 3B, Table S4). Accessibility refers to the ease of travel to and from a city [35] and urban expansion often occurs in densely forested areas with high accessibility. In Ethiopia, healthcare accessibility had a significant association with the national distribution of HIV, TB, and malaria [50]. Moreover, previous spatial malaria models extensively used accessibility to cities, as it is believed to be inversely related to the probability of a patient seeking treatment at a health facility [51]. Moreover, higher population density promotes virus transmission and increases the likelihood of animal–human interactions. The mortality rate reflects the local infection situation and healthcare capacity. Direct contact with infected humans, such as animal keepers and family members, represents the primary route of animal infection [26]. The XGBoost model reveals distinct patterns regarding landscape and climatic factors compared to other models. While landscape characteristics show relatively low individual importance in XGBoost (most variables < 3%), climatic conditions demonstrate notably higher contributions compared to BRT and RF models, with precipitation (5.0%), wind speed (5.0%), solar radiation (4.0%), and water vapor pressure (4.0%; Figure 3B, Table S4). This suggests that the XGBoost algorithm captures climate-driven transmission pathways more effectively than other models, indicating the potential importance of environmental conditions in virus spread that previous studies have recognized. Urbanization and land development have heightened the frequency of human–wildlife encounters, posing a risk of wildlife infection through contaminated wastewater [6]. The SARS-CoV-2 virus can be transmitted via environmental media, indicating the possibility of wildlife infection through human discharge of virus-containing pollutants [6].

High densities of livestock species, such as ducks and pigs, have been identified as significant factors, highlighting the critical importance of addressing the transmission of SARS-CoV-2 in livestock farming. A study found a strong positive correlation between livestock processing plants and the spread of COVID-19 within local communities, suggesting that these facilities may contribute to superspreader events in the human population, facilitating the transmission of the virus to nearby populations [52]. However, the risks associated with livestock processing plants only affected human infections, and the association with animal infections was not significant. Infections have been documented in ferret and mink farms in Denmark, Greece, Lithuania, the Netherlands, Spain, Sweden, and the USA. Feral cats residing in the vicinity of farms have also tested positive for the virus [53]. However, while existing global distribution data on livestock allowed us to establish fundamental evidence, more detailed data are required to investigate factors such as feeding practices, animal movements, and the use of feed and drugs, all of which can contribute to increased animal infections [54].

Based on the intricate impacts of these variables, our models identify areas at elevated risk for SARS-CoV-2 infections in animals, including southern North America, Europe, Uruguay, Argentina, Brazil, China, India, and Japan. These results emphasize the importance of intensifying disease surveillance in both wild and domestic animal populations. While most of Africa showed low-risk levels, monitoring was warranted in some coastal regions of the Gulf of Guinea, South Africa, and a few high-risk areas. Based on these findings, we recommend prioritizing animal health surveillance in the high-risk areas of Western and Central Europe, Southern Asia, the Eastern United States, and Southern Brazil. Although China, India, and Brazil have reported a limited number of animal infection cases, the predicted values suggest a high-risk level warranting attention (Table S6). The high population densities and relative high accessibility in these countries may increase the likelihood of SARS-CoV-2 spillback events involving animals. In contrast, our overall assessment for the African region indicates a lower risk, attributable to limited regional accessibility, factors that discourage SARS-CoV-2 transmission. The substantial proportion of high-risk areas within Belgium, Bangladesh, Germany, and Switzerland raises concerns about the well-being of local animal populations. These European countries exhibit very high-risk areas likely due to their high population densities, excellent urban accessibility, extensive transportation networks, and intensive livestock farming practices, particularly mink farming operations that have historically reported significant SARS-CoV-2 outbreaks. Additionally, these regions' advanced healthcare systems and research infrastructure may contribute to higher detection rates, while their dense human–animal interface in both agricultural and urban settings increases transmission opportunities. However, the impact of SARS-CoV-2 on animal health and welfare is relatively limited, except for mink.

Our findings demonstrate that anthropogenic factors have greater predictive capabilities for animal infections compared to the biophysical factors examined in this study, suggesting potential benefits may be achieved through synergies between public health and animal health policies. The concept of One Health, promoted by WHO, WOAH, FAO, and UNEP, should be emphasized as a key approach in managing emerging zoonotic diseases, particularly for source prevention. Animal SARS-CoV-2 infections are closely linked to the severity of human COVID-19 [55]. Close collaboration and information sharing between the public health and animal health sectors can lead to integrated policy measures that reduce human–animal contact and transmission, thereby preventing the spread of disease between human and animal populations. This synergy can result in increased vaccination rates, improved biosecurity measures, animal husbandry and management policies, and enhanced surveillance and response strategies to effectively prevent and control animal infections. Policymakers and stakeholders must consider the impact of human activities when formulating animal health policies, and vice versa. There is evidence to suggest that neglecting human factors may result in inadequate policy development for the prevention and control of animal infections [56]. Therefore, prioritizing the impact of human activities on animal infections and striving for synergy between public health and animal health through comprehensive policy development is essential.

## 5. Limitation

It is important to emphasize that the models employed in this study aimed to estimate the likelihood of more frequent occurrences or future emergence of SARS-CoV-2 infections in animals. The model was trained using data-rich regions such as the Americas and Europe to predict the risk of SARS-CoV-2 infection in animals. However, when expanding the model to map global risk, its predictions may be inaccurate for African and Asian regions. The model has not yet found suitable data to assess the environmental and frequency factors of human–animal contact. Furthermore, it has not considered the possibility of virus circulation and/or variation within species, including the concept of some animal species, such as dogs, serving as dead-end hosts. In summary, our results reinforce the importance of ongoing animal health surveillance and preparedness efforts aimed at mitigating the emergence and spread of future zoonotic diseases.

Another important limitation concerns our species selection approach, which focused exclusively on IUCN Red List species for habitat distribution analysis. This approach inadvertently excluded commercially important species that may represent significant epidemiological risks, particularly in China and Southeast Asia. Raccoon dogs, pangolins (as documented in our infection dataset), and various farmed mustelids are central to wildlife trade networks and farming operations but lack standardized habitat distribution data. The absence of systematic surveillance in these species, combined with their presence in wet markets and high-density farming operations, represents a critical knowledge gap. Future studies should prioritize mapping commercial farming distributions and trade routes for these species, integrating agricultural and wildlife farming registry data to better assess spillover risk in regions with intensive animal–human interfaces.

Our analysis also did not explicitly model the spatial distribution of domestic animals (cats, dogs, hamsters), despite their documented susceptibility and substantial representation in our infection dataset. While we considered incorporating pet animals, reliable global datasets for domestic animal distributions are not available with the spatial resolution and standardization required for our modeling approach. Additionally, domestic animal distributions are strongly correlated with human population density, which was identified as the second most important predictor in our models (31.83% importance in BRT, 14.30% in RF), suggesting our analysis may indirectly capture some aspects of domestic animal exposure risk. However, this approach cannot account for regional variations in pet ownership patterns, stray animal populations, or the specific spatial dynamics of pet-wildlife interface zones, which represent important knowledge gaps for understanding bidirectional spillover risk between domestic animals and wildlife populations. Our analysis also lacked comprehensive bat species mapping, despite their role as natural coronavirus reservoirs. Limited bat infection data in our dataset precluded robust modeling for this group. Future studies should integrate bat distribution data with anthropogenic risk factors to identify potential spillover hotspots where bat–human interfaces coincide with favorable viral emergence conditions. Our analysis focused primarily on human-to-animal transmission pathways and did not explicitly model potential virus transmission among susceptible animals sharing overlapping habitats. Interspecies transmission through shared environmental resources (such as water sources) or direct contact represents an important knowledge gap, particularly for species pairs like American mink and white-tailed deer that may share geographic areas and common resources. Future research should integrate species behavioral ecology data, environmental persistence characteristics, and contact probability matrices to model interspecies transmission networks and identify habitat overlap zones, where cross-species transmission risk may be elevated.

## 6. Conclusion

Harnessing recent advancements in ML for predictive spatial mapping can provide valuable insights into the unexplored distribution of SARS-CoV-2 in animals. The risk map generated from these models serves as a crucial tool for surveillance and the implementation of preventive measures. However, further research is required to validate the findings and establish potential causal relationships. As more data on SARS-CoV-2 infections in animals accumulates, these models can be refined to offer more accurate predictions, facilitating informed decision-making for virus control.

## Figures and Tables

**Table 1:** List of drivers and their corresponding data sources.

Variable	Source	Note	Period
Climatic conditions	WorldClim, version 2.1	This dataset was released in January 2020 and includes variables such as wind speed (m s^−1^), water vapor pressure (kPa), minimum temperature (°C), maximum temperature (°C), average temperature (°C), precipitation (mm), and solar radiation (kJ m^−2^ day^−1^) at a 2.5 arc-minute resolution	1970–2020
Landscape characteristics	Copernicus global land service (100 m)	A 100 m resolution raster digital map was used to calculate Shannon's diversity index (SHDI), number of patches (NP), edge density (ED), landscape shape index (LSI), largest patch index (LPI), patch density (PD), and mean patch fractal dimension (MPFD)	2019
Livestock density	Gilbert et al. [32]	The data source includes the density of cattle, buffaloes, horses, sheep, goats, pigs, chickens, and ducks at a 5 arc-minute resolution	2010
Population density	The gridded population of the world, version 4 (GPWv4)	The dataset utilized a proportional allocation gridding algorithm, employing approximately 13.5 million national and subnational administrative units, to assign UN WPP-adjusted population counts to 30 arc-second grid cells	2020
Biodiversity	Newbold et al. [33]	The Biodiversity Intactness Index represents the modeled average abundance of species that were originally present, relative to their abundance in an intact ecosystem	2016
Susceptible animals distribution	The IUCN red list of threatened species	The data collection focused on the distribution of the following species: American mink (*Neovison vison*), Black-footed Ferret (*Mustela nigripes*), Western Gorilla (*Gorilla gorilla*), Lion (*Panthera leo*), Snow Leopard (*Panthera uncia*), Tiger (*Panthera tigris*), White-tailed Deer (*Odocoileus virginianus*), Asian Small-clawed Otter (*Aonyx cinereus*), and Puma (*Puma concolor*)	2015
COVID-19 mortality	Xu et al. [34]	The dataset provides information on the mortality rate, which is calculated as the number of deaths divided by the local population.	2020
Accessibility	Weiss et al. [35]	The dataset quantifies travel time to cities in 2015 at a spatial resolution of approximately 1 km by 1 km	2015

## Data Availability

The data that support the findings of this study are openly available in figshare at https://figshare.com/s/8df40ccaa4fd00780650.

## Supporting Information

Supporting Information Figure S1. Generation process for georeferenced dataset of SARS-CoV-2 infections in animals. Figure S2. Projection of SARS-CoV-2 infection risk in animals. Figure S3. Mapping the distribution of moderate to high-risk areas in habitats of susceptible animals. (A) American mink (*Neovison vison*); (B) Puma (*Puma concolor*); (C) White-tailed Deer (*Odocoileus virginianus*); (D) Western Gorilla (*Gorilla gorilla*); (E) Lion (*Panthera leoPanthera leo*); (F) Asian Small-clawed Otter (*Aonyx cinereus*); (G) Snow Leopard (*Panthera uncia*); (H) Tiger (*Panthera tigris*); (I) Black-footed Ferret (*Mustela nigripes*). Table S1. Summary of countries with the highest reported cases of SARS-CoV-2 infections in animals worldwide. Table S2. Distribution of high-risk areas in habitats of susceptible animals, categorized by country. Table S3. Categorization of areas by country based on projected levels of risk. Table S4. Comparison of the capabilities of 27 variables as drivers of SARS-CoV-2 infection in animals across three models. Table S5. Categorization of susceptible species distribution by country at different risk levels. Table S6. Distribution of high-risk areas in habitats of susceptible animals, categorized by country.

## References

[1] Lu R. J., Zhao X., Li J. (2020). Genomic Characterisation and Epidemiology of 2019 Novel Coronavirus: Implications for Virus Origins and Receptor Binding. *The Lancet*.

[2] Andersen K. G., Rambaut A., Lipkin W. I., Holmes E. C., Garry R. F. (2020). The Proximal Origin of SARS-CoV-2. *Nature Medicine*.

[3] Garigliany M., Van Laere A. S., Clercx C. (2020). SARS-CoV-2 Natural Transmission From Human to Cat, Belgium, March 2020. *Emerging Infectious Diseases*.

[4] McAloose D., Laverack M., Wang L. Y. (2020). From People to *Panthera*: Natural SARS-CoV-2 Infection in Tigers and Lions at the Bronx Zoo. *mBio*.

[5] Gortazar C., Barroso-Arevalo S., Ferreras-Colino E. (2021). Natural SARS-CoV-2 Infection in Kept Ferrets, Spain. *Emerging Infectious Diseases*.

[6] Aguilo-Gisbert J., Padilla-Blanco M., Lizana V. (2021). First Description of SARS-CoV-2 Infection in Two Feral American Mink (*Neovison vison*) Caught in the Wild. *Animals-Basel*.

[7] Chandler J. C., Bevins S. N., Ellis J. W. (2021). SARS-CoV-2 Exposure in Wild White-Tailed Deer (*Odocoileus virginianus*). *Proceedings of the National Academy of Sciences*.

[8] Hale V. L., Dennis P. M., McBride D. S. (2022). SARS-CoV-2 Infection in Free-Ranging White-Tailed Deer. *Nature*.

[9] Kuchipudi S. V., Surendran-Nair M., Ruden R. M. (2022). Multiple Spillovers From Humans and Onward Transmission of SARS-CoV-2 in White-Tailed Deer. *Proceedings of the National Academy of Sciences*.

[10] Kok K. H., Wong S. C., Chan W. M. (2022). Co-Circulation of Two SARS-CoV-2 Variant Strains Within Imported Pet Hamsters in Hong Kong. *Emerging Microbes & Infections*.

[11] Patterson E. I., Elia G., Grassi A. (2020). Evidence of Exposure to SARS-CoV-2 in Cats and Dogs From Households in Italy. *Nature Communications*.

[12] Oude Munnink B. B., Sikkema R. S., Nieuwenhuijse D. F. (2021). Transmission of SARS-CoV-2 on Mink Farms Between Humans and Mink and Back to Humans. *Science*.

[13] Hammer A. S., Quaade M. L., Rasmussen T. B. (2021). SARS-CoV-2 Transmission Between Mink (*Neovison vison*) and Humans, Denmark. *Emerging Infectious Diseases*.

[14] Larsen H. D., Fonager J., Lomholt F. K. (2021). Preliminary Report of an Outbreak of SARS-CoV-2 in Mink and Mink Farmers Associated With Community Spread, Denmark, June to November 2020. *Euro Surveill*.

[15] Fenollar F., Mediannikov O., Maurin M. (2021). SARS-CoV-2, and the Human-Animal Interface. *Frontiers in Microbiology*.

[16] Koopmans M. (2021). SARS-CoV-2 and the Human-Animal Interface: Outbreaks on Mink Farms. *The Lancet Infectious Diseases*.

[17] Plowright R. K., Reaser J. K., Locke H. (2021). Land use-Induced Spillover: A Call to Action to Safeguard Environmental, Animal, and Human Health. *The Lancet Planetary Health*.

[18] Patz J. A., Daszak P., Tabor G. M. (2004). Unhealthy Landscapes: Policy Recommendations on Land use Change and Infectious Disease Emergence. *Environmental Health Perspectives*.

[19] Faust C. L., McCallum H. I., Bloomfield L. S. P. (2018). Pathogen Spillover During Land Conversion. *Ecology Letters*.

[20] Kubota Y., Shiono T., Kusumoto B., Fujinuma J. (2020). Multiple Drivers of the COVID-19 Spread: The Roles of Climate, International Mobility, and Region-Specific Conditions. *PLoS ONE*.

[21] Ma Y. L., Zhao Y. D., Liu J. T. (2020). Effects of Temperature Variation and Humidity on the Death of COVID-19 in Wuhan, China. *Science of The Total Environment*.

[22] Ahlawat A., Wiedensohler A., Mishra S. K. (2020). An Overview on the Role of Relative Humidity in Airborne Transmission of SARS-CoV-2 in Indoor Environments. *Aerosol and Air Quality Research*.

[23] Tateo F., Fiorino S., Peruzzo L. (2022). Effects of Environmental Parameters and Their Interactions on the Spreading of SARS-CoV-2 in North Italy Under Different Social Restrictions. A New Approach Based on Multivariate Analysis. *Environmental Research*.

[24] Sagripanti J. L., Lytle C. D. (2020). Estimated Inactivation of Coronaviruses by Solar Radiation With Special Reference to COVID-19. *Photochemistry and Photobiology*.

[25] Ahmadi M., Sharifi A., Dorosti S., Jafarzadeh Ghoushchi S., Ghanbari N. (2020). Investigation of Effective Climatology Parameters on COVID-19 Outbreak in Iran. *Science of the Total Environment*.

[26] Chen J. (2020). Pathogenicity and Transmissibility of 2019-nCoV—A Quick Overview and Comparison With Other Emerging Viruses. *Microbes and Infection*.

[27] Larsen H. D., Fonager J., Lomholt F. K. (2021). Preliminary Report of an Outbreak of SARS-CoV-2 in Mink and Mink Farmers Associated With Community Spread, Denmark, June to November 2020. *Eurosurveillance*.

[28] Sundaram M. E., Calzavara A., Mishra S. (2021). Individual and Social Determinants of SARS-CoV-2 Testing and Positivity in Ontario, Canada: A Population-Wide Study. *Canadian Medical Association Journal*.

[29] Jia Z., Lu Z. (2020). Modelling COVID-19 Transmission: From Data to Intervention. *The Lancet Infectious Diseases*.

[30] Zhang G., Zheng D., Tian Y., Li S. (2019). A Dataset of Distribution and Diversity of Ticks in China. *Scientific Data*.

[31] Fang R., Yang X., Guo Y. (2024). SARS-CoV-2 Infection in Animals: Patterns, Transmission Routes, and Drivers. *Eco-Environment & Health*.

[32] Gilbert M., Nicolas G., Cinardi G. (2018). Global Distribution Data for Cattle, Buffaloes, Horses, Sheep, Goats, Pigs, Chickens and Ducks in 2010. *Scientific Data*.

[33] Newbold T., Hudson L. N., Arnell A. (2016). Global Map of the Biodiversity Intactness Index. *Environmental Science*.

[34] Xu B., Gutierrez B., Mekaru S. (2020). Epidemiological Data From the COVID-19 Outbreak, Real-Time Case Information. *Scientific Data*.

[35] Weiss D. J., Nelson A., Gibson H. S. (2018). A Global Map of Travel Time to Cities to Assess Inequalities in Accessibility in 2015. *Nature*.

[36] Fang L. Q., Li X. L., Liu K. (2013). Mapping Spread and Risk of Avian Influenza A (H7N9) in China. *Scientific Reports*.

[37] Martin V., Pfeiffer D. U., Zhou X. Y. (2011). Spatial Distribution and Risk Factors of Highly Pathogenic Avian Influenza (HPAI) H5N1 in China. *PLoS Pathogens*.

[38] Farooq Z., Rocklov J., Wallin J. (2022). Artificial Intelligence to Predict West Nile Virus Outbreaks With Eco-Climatic Drivers. *The Lancet Regional Health - Europe*.

[39] Crockett P. F., Keough M. J. (2014). Ecological Niches of Three Abundant Caulerpa Species in Port Phillip Bay, Southeast Australia. *Aquatic Botany*.

[40] Kraemer M. U. G., Golding N., Brady O. J. (2015). The Global Distribution of the Arbovirus Vectors *Aedes aegypti* and Ae. albopictus. *eLife*.

[41] Elith J., Graham C. H., Anderson R. P. (2006). Novel Methods Improve Prediction of Species’ Distributions From Occurrence Data. *Ecography*.

[42] Elith J., Leathwick J. R., Hastie T. (2008). A Working Guide to Boosted Regression Trees. *Journal of Animal Ecology*.

[43] Pan B. (2018). Application of XGBoost Algorithm in Hourly PM2.5 Concentration Prediction. *IOP Conference Series: Earth and Environmental Science*.

[44] Ibrahem Ahmed Osman A., Najah Ahmed A., Chow M. F., Feng Huang Y., El-Shafie A. (2021). Extreme Gradient Boosting (Xgboost) Model to Predict the Groundwater Levels in Selangor Malaysia. *Ain Shams Engineering Journal*.

[45] Breiman L. (2001). Random Forests. *Machine Learning*.

[46] Barbet-Massin M., Jiguet F., Albert C. H., Thuiller W. (2012). Selecting Pseudo-Absences for Species Distribution Models: How, Where and How Many?. *Methods in Ecology and Evolution*.

[47] McPherson J. M., Jetz W., Rogers D. J. (2004). The Effects of Species’ Range Sizes on the Accuracy of Distribution Models: Ecological Phenomenon or Statistical Artefact?. *Journal of Applied Ecology*.

[48] Marmion M., Luoto M., Heikkinen R. K., Thuiller W. (2009). The Performance of State-of-the-Art Modelling Techniques Depends on Geographical Distribution of Species. *Ecological Modelling*.

[49] Lefrancois T., Malvy D., Atlani-Duault L. (2023). After 2 Years of the COVID-19 Pandemic, Translating One Health into Action Is Urgent. *The Lancet*.

[50] Alene K. A., Elagali A., Barth D. D. (2022). Spatial Codistribution of HIV, Tuberculosis and Malaria in Ethiopia. *BMJ Global Health*.

[51] Elagali A., Ahmed A., Makki N. (2022). Spatiotemporal Mapping of Malaria Incidence in Sudan Using Routine Surveillance Data. *Scientific Reports*.

[52] Taylor C. A., Boulos C., Almond D. (2020). Livestock Plants and COVID-19 Transmission. *Proceedings of the National Academy of Sciences*.

[53] van Aart A. E., Velkers F. C., Fischer E. A. J. (2022). SARS-CoV-2 Infection in Cats and Dogs in Infected Mink Farms. *Transboundary and Emerging Diseases*.

[54] Haque M. H., Islam M. A., Karim M. R. (2021). Coronavirus Disease 2019 and Future Pandemics: Impacts on Livestock Health and Production and Possible Mitigation Measures. *Veterinary World*.

[55] Saegerman C., Bianchini J., Renault V., Haddad N., Humblet M. F. (2021). First Expert Elicitation of Knowledge on Drivers of Emergence of the COVID-19 in Pets. *Transboundary and Emerging Diseases*.

[56] Magouras I., Brookes V. J., Jori F., Martin A., Pfeiffer D. U., Durr S. (2020). Emerging Zoonotic Diseases: Should We Rethink the Animal–Human Interface?. *Frontiers in Veterinary Science*.

