# Supplementary material for: Predictive Modeling of Global SARS-CoV-2 Infection Risk in Animals: Unveiling Potential Reservoirs and Informing Health Policy Synergies
Source: Transbound Emerg Dis. 2025 Sep 30;2025:3959370. doi: 10.1155/tbed/3959370 (PMC12503988; doi:10.1155/tbed/3959370)
Supplement: Supporting Information — Figure S1. Generation process for georeferenced dataset of SARS-CoV-2 infections in animals. Figure S2. Projection of SARS-CoV-2 infection risk in animals. Figure S3. Mapping the distribution of moderate to high-risk areas in habitats of susceptible animals. (A) American mink (Neovison vison); (B) Puma (Puma concolor); (C) White-tailed Deer (Odocoileus virginianus); (D) Western Gorilla (Gorilla gorilla); (E) Lion (Panthera leoPanthera leo); (F) Asian Small-clawed Otter (Aonyx cinereus); (G) Snow Leopard (Panthera uncia); (H) Tiger (Panthera tigris); (I) Black-footed Ferret (Mustela nigripes). Table S1. Summary of countries with the highest reported cases of SARS-CoV-2 infections in animals worldwide. Table S2. Distribution of high-risk areas in habitats of susceptible animals, categorized by country. Table S3. Categorization of areas by country based on projected levels of risk. Table S4. Comparison of the capabilities of 27 variables as drivers of SARS-CoV-2 infection in animals across three models. Table S5. Categorization of susceptible species distribution by country at different risk levels. Table S6. Distribution of high-risk areas in habitats of susceptible animals, categorized by country. [file 3959370.f1.docx]

**Supplemental Figures and Tables**

**Figure S1.** Generation process for georeferenced dataset of SARS-CoV-2 infections in animals.

**Figure S2.** Projection of SARS-CoV-2 infection risk in animals.

**Figure S3.** Mapping the distribution of moderate to high-risk areas in habitats of susceptible animals. (A) American mink (*Neovison vison*); (B) Puma (*Puma concolor*); (C) White-tailed Deer (*Odocoileus virginianus*); (D) Western Gorilla (*Gorilla gorilla*); (E) Lion (*Panthera leoPanthera leo*); (F) Asian Small-clawed Otter (*Aonyx cinereus*); (G) Snow Leopard (*Panthera uncia*); (H) Tiger (*Panthera tigris*); (I) Black-footed Ferret (*Mustela nigripes*).

**Table S1.** Summary of countries with the highest reported cases of SARS-CoV-2 infections in animals worldwide.

**Table S2.** Distribution of high-risk areas in habitats of susceptible animals, categorized by country.

**Table S3.** Categorization of areas by country based on projected levels of risk.

**Table S4.** Comparison of the capabilities of 27 variables as drivers of SARS-CoV-2 infection in animals across three models.

**Table S5.** Categorization of susceptible species distribution by country at different risk levels.

**Table S6.** Distribution of high-risk areas in habitats of susceptible animals, categorized by country.

**Figure S1:** Generation process for georeferenced dataset of SARS-CoV-2 infections in animals.


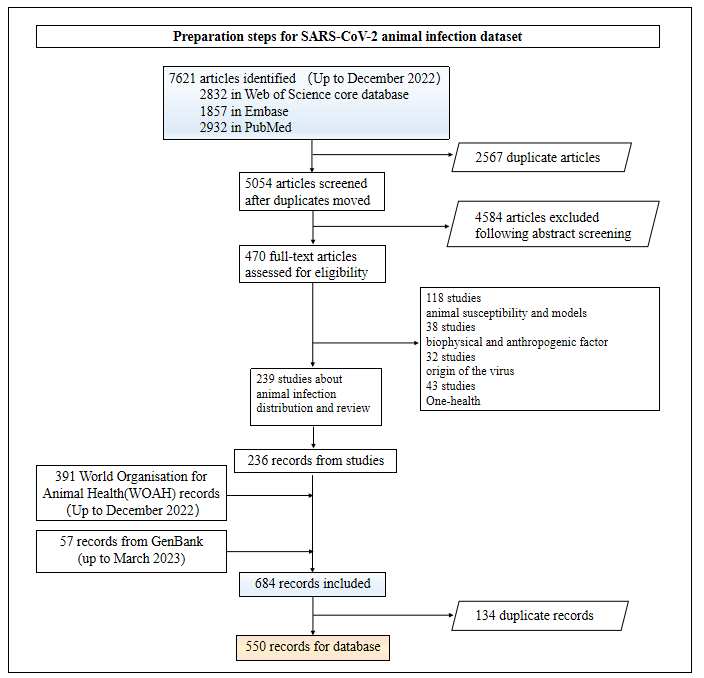


**Figure S2:** Projection of SARS-CoV-2 infection risk in animals.

**(A)** Boosted regression tree (BRT)


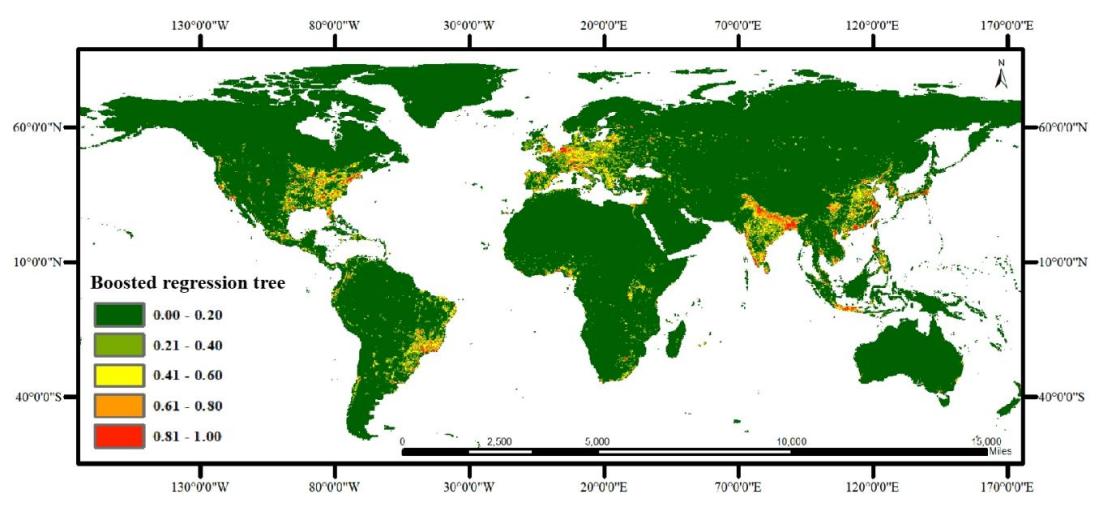


**(B)** eXtreme gradient boosting (XGBoost)


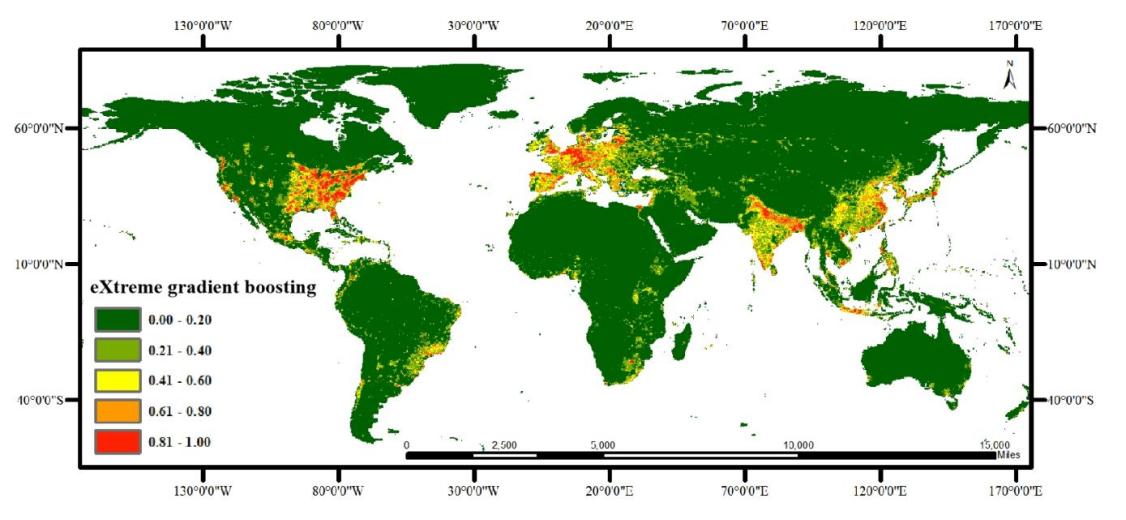


**(C)** Random forests (RF)


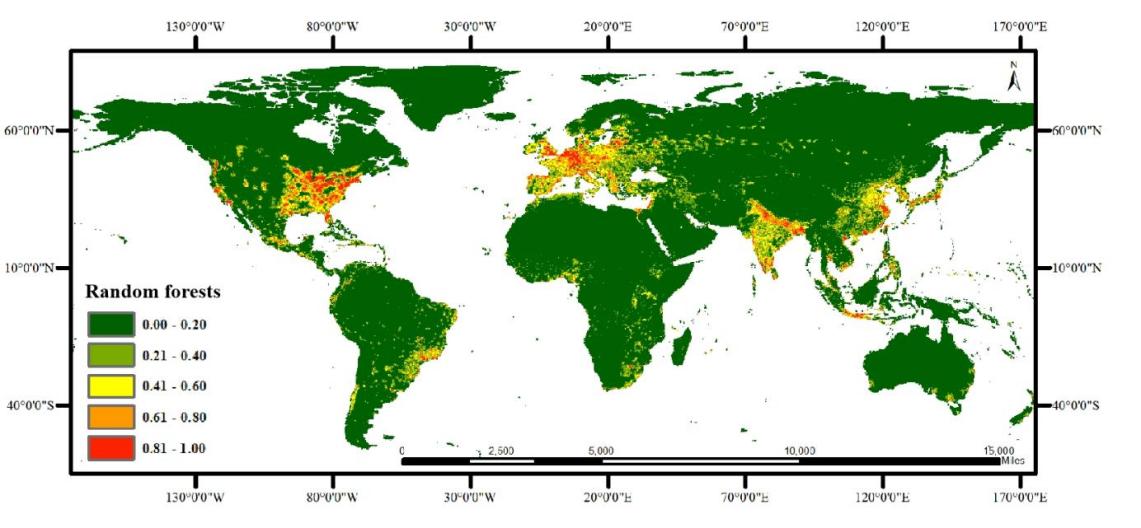


**Figure S3:** Mapping the distribution of moderate to high-risk areas in habitats of susceptible animals.

**(A)** American mink (*Neovison vison*)


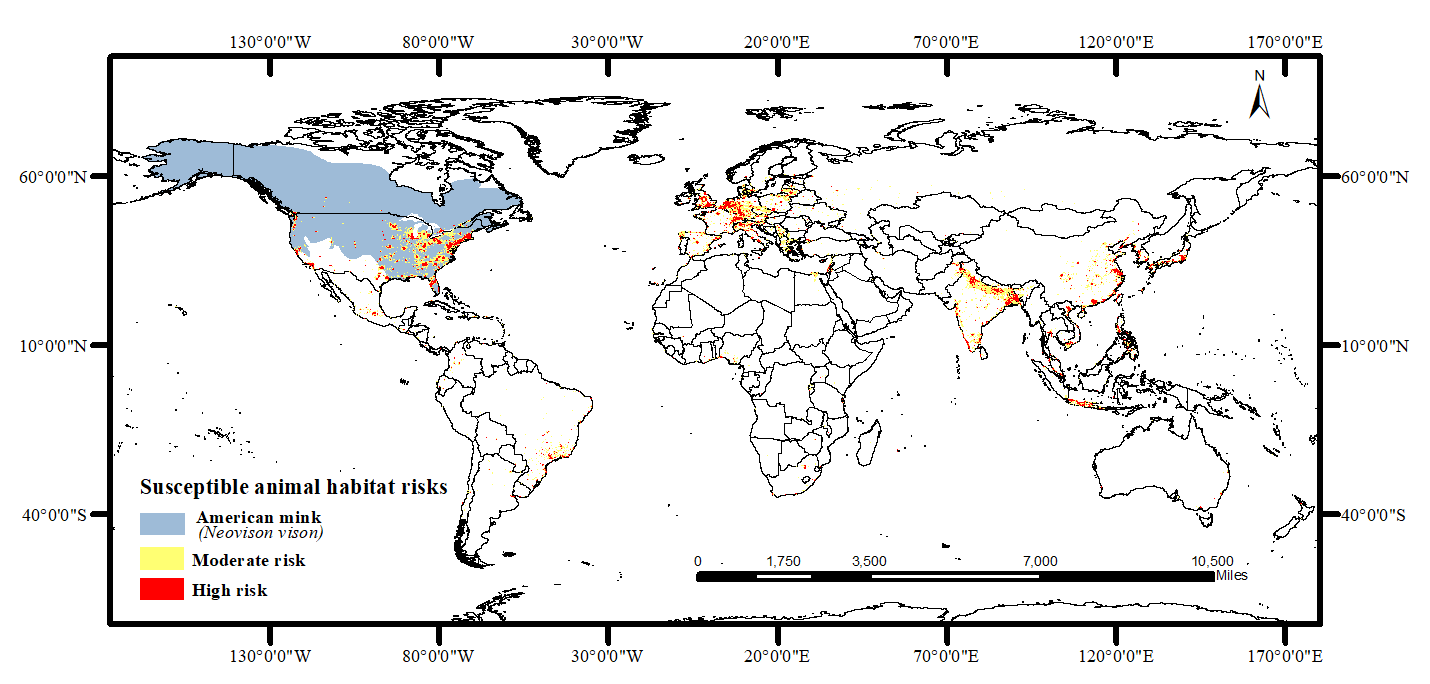


**(B)** Puma (*Puma concolor*)


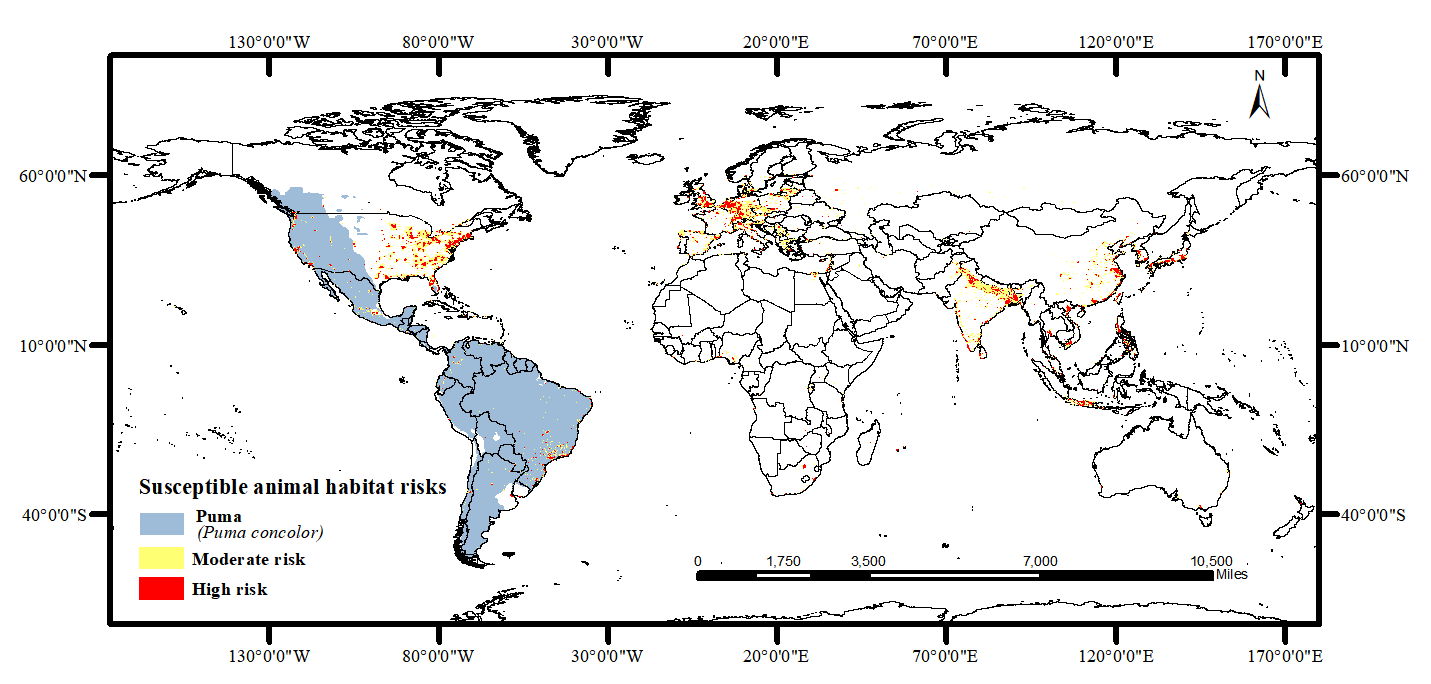


**(C)** White-tailed Deer (*Odocoileus virginianus*)


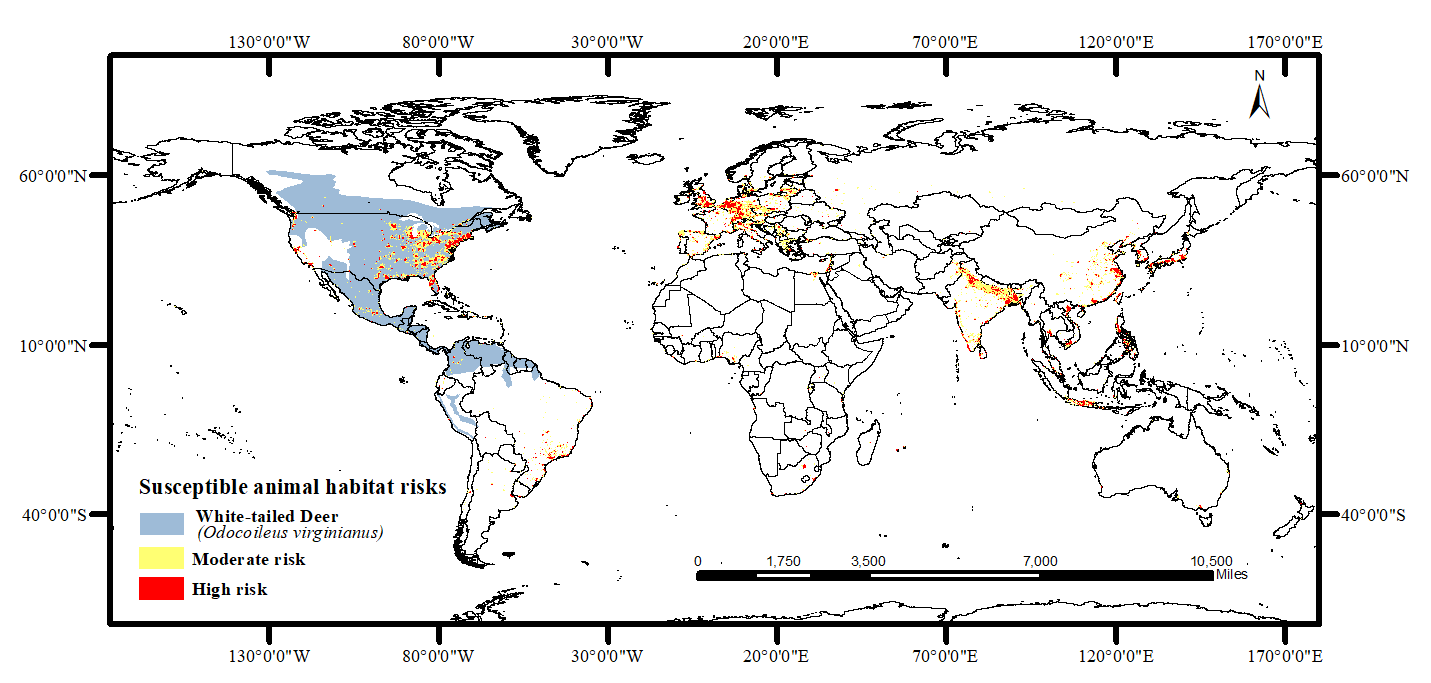


**(D)** Western Gorilla (*Gorilla gorilla*)


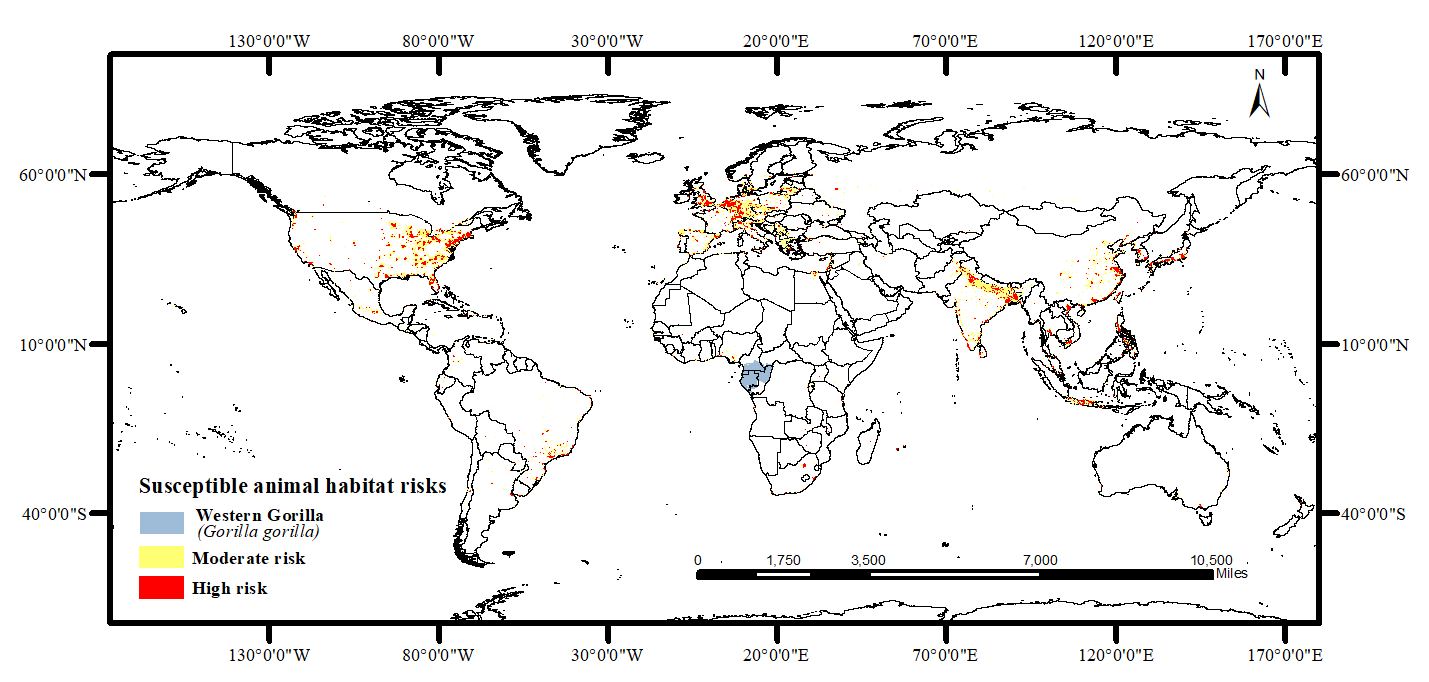


**(E)** Lion (*Panthera leoPanthera leo*)


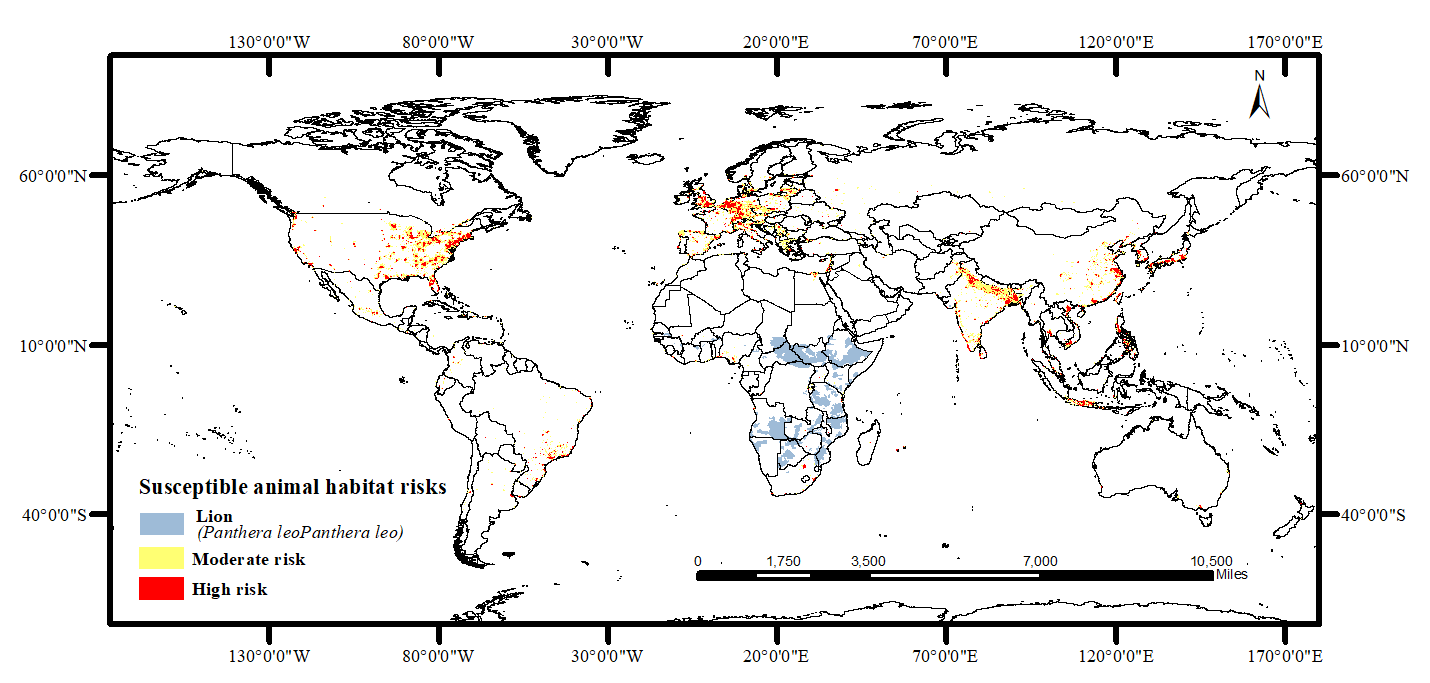


**(F)** Asian Small-clawed Otter (*Aonyx cinereus*)


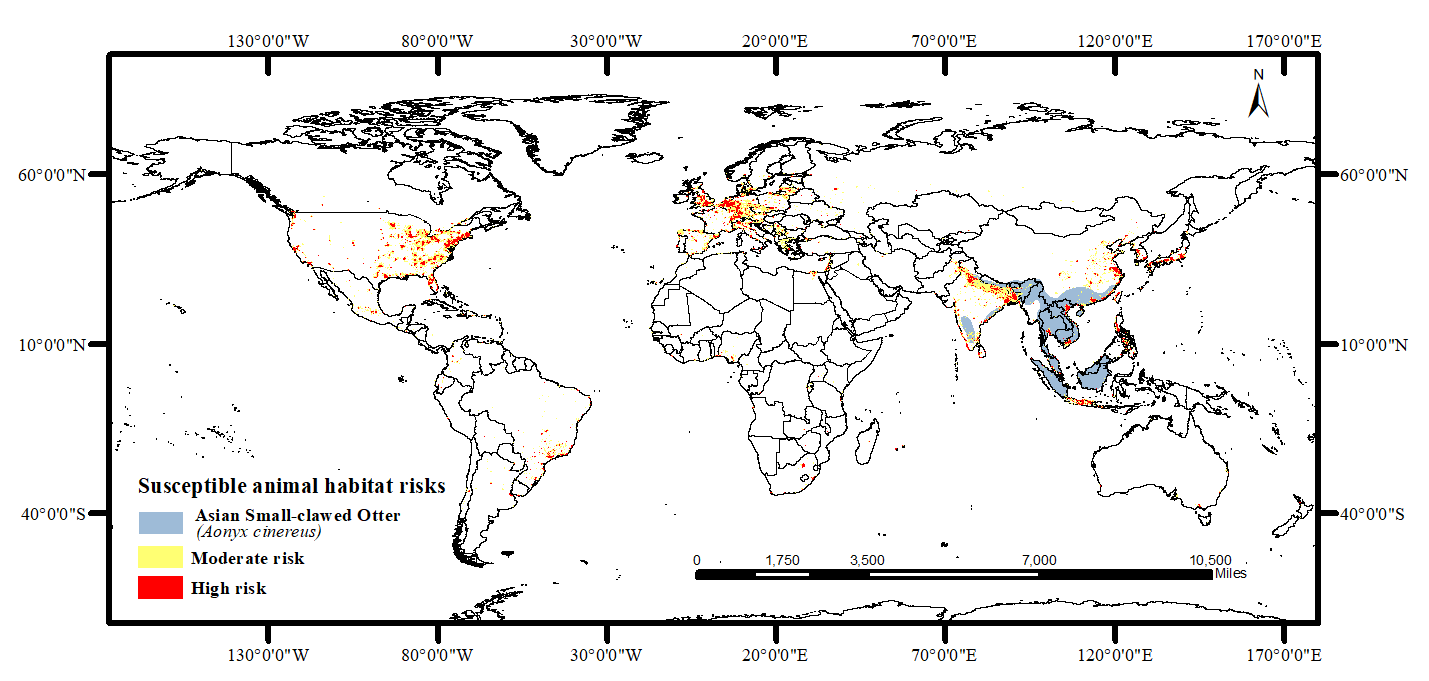


**(G)** Snow Leopard (*Panthera uncia*)


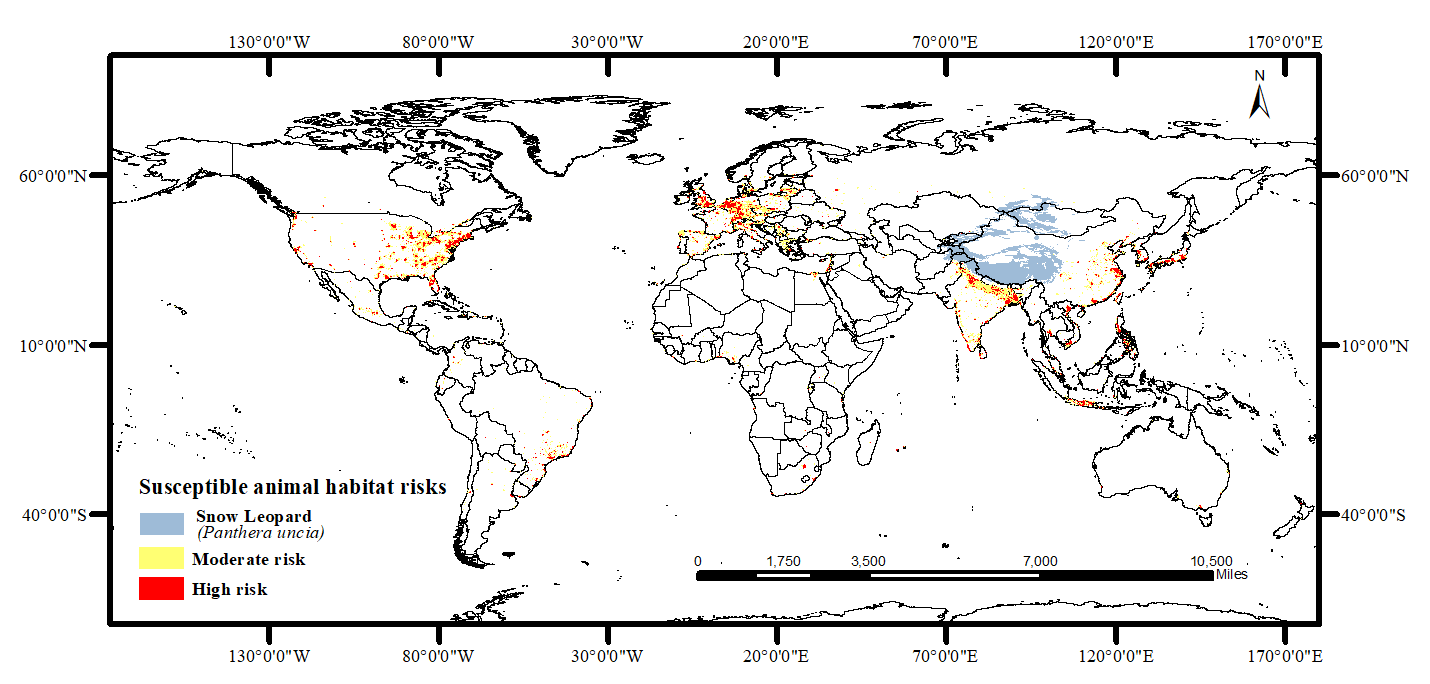


**(H)** Tiger (*Panthera tigris*)


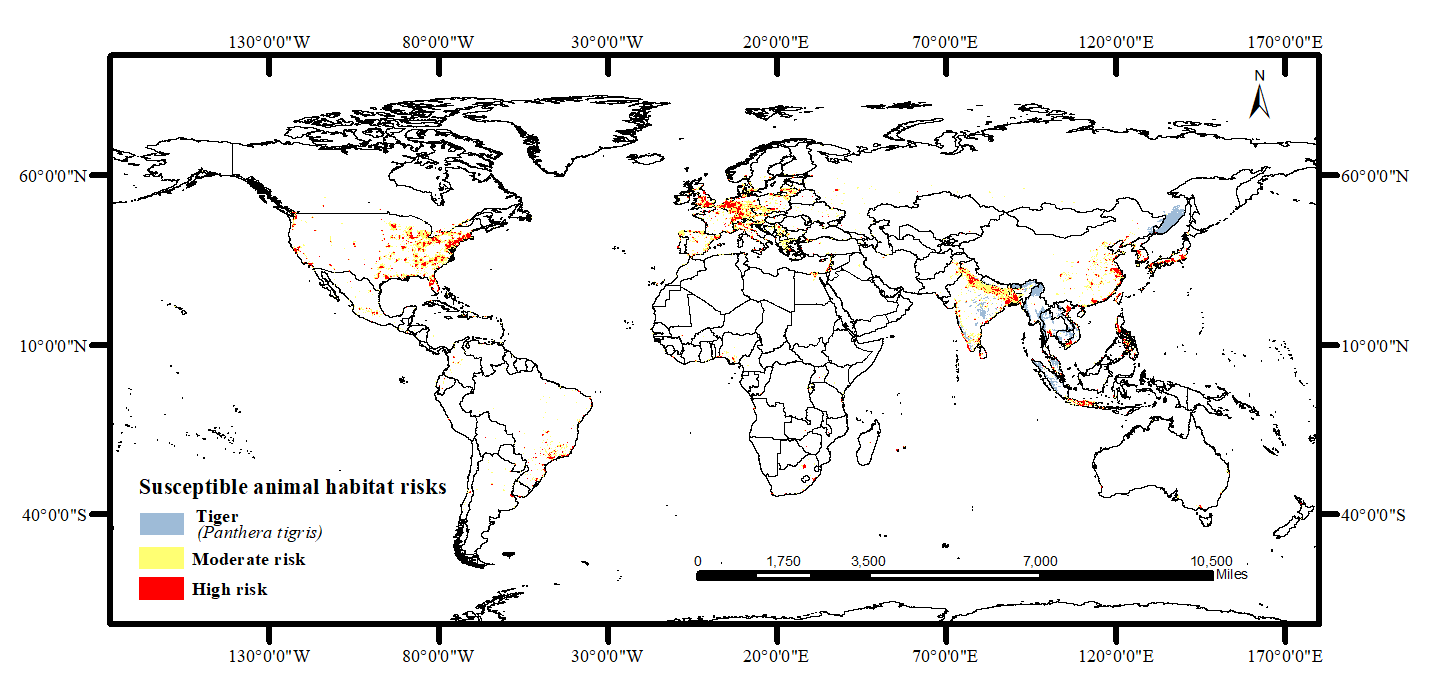


**(I)** Black-footed Ferret (*Mustela nigripes*)


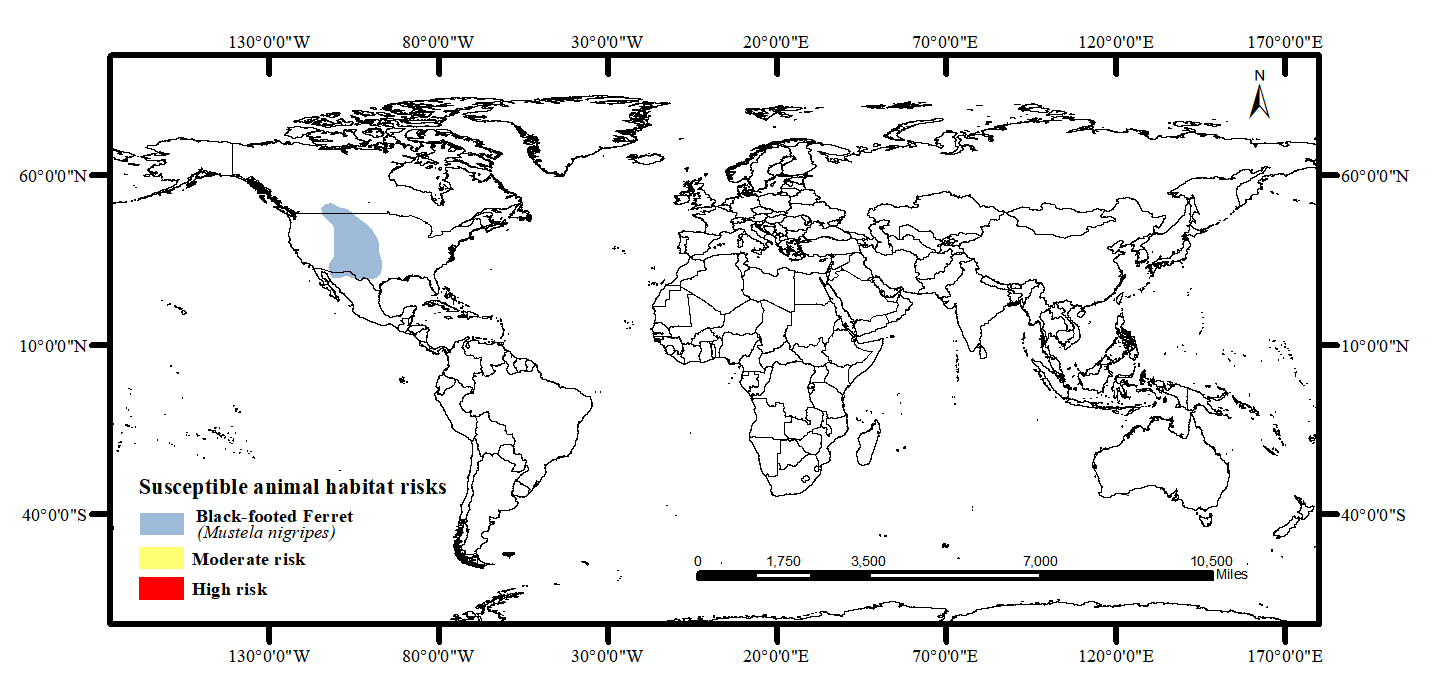


**Table S1:** Summary of countries with the highest reported cases of SARS-CoV-2 infections in animals worldwide.

| **Country** | **Species number** | **Sum reports** | **Animals (reports)** | **Sum cases** | **Animals (cases)** |
| --- | --- | --- | --- | --- | --- |
| Greece | 3 | 19 | Cat(2) Mink(4) Mustelidae(13) | 15642 | Cat(4) Mink(12) Mustelidae(15626) |
| Netherlands | 4 | 22 | Cat(1) Dog(1) Mink(20) | 9038 | Cat(2) Dog(1) Mink(9035) |
| United States of America | 18 | 181 | Binturongs(2) Canadian lynxes(1) Cat(57) Dog(37)  Mink(21) Gorilla(5) Lion(11) Mustelidae(1) Otter(6) Puma(1) Fishing cat(2) Snow Leopards(6) South American Coatis(2) Hyena(4) Spotted Hyenas(2) Tiger(22) White-Tailed deer(8) Asian small-clawed otter(3) South American Coati(2) | 569 | Binturongs(2) Canadian lynxes(1) Cat(60) Dog(39)  Mink(42) Gorilla(16) Lion(28) Mustelidae(1) Otter(6) Puma(1) Fishing cat(2) Snow Leopards(13) South American Coatis(2) Hyena(4) Spotted Hyenas(2) Tiger(28) White-Tailed deer(312) Asian small-clawed otter(7) South American Coati(2) |
| Lithuania | 2 | 17 | Mink(16) Mustelidae(1) | 543 | Mink(538) Mustelidae(5) |
| Canada | 5 | 11 | Cat(3) Dog(1) Mustelidae(3) White-Tailed deer(4) | 284 | Cat(3) Dog(1) Mustelidae(253) White-Tailed deer(27) |
| Spain | 6 | 33 | Cat(11) Gorilla(1) Lion(1) Mink(14) Ferret(1) Dog(5) | 205 | Cat(54) Gorilla(5) Lion(4) Mink(132) Ferret(1) Dog(9) |
| Poland | 4 | 9 | Cat(2) Dog(2) Mustelidae(3) Mink(2) | 120 | Cat(46) Dog(62) Mustelidae(10) Mink(2) |
| Thailand | 4 | 30 | Bat(2) Cat(20) Dog(7) Gray wolf(1) | 81 | Bat(13) Cat(26) Dog(41) Gray wolf(1) |
| Egypt | 2 | 4 | Cat(2) Dog(2) | 66 | Cat(33) Dog(33) |
| China | 4 | 6 | Cat(2) Cricetidae(1) Dog(2) pangolins(1) | 62 | Cat(27) Cricetidae(15) Dog(17) pangolins(3) |

Detailed reference information for all studies contributing to the infection data presented in this table is available in our publicly accessible dataset: "A dataset of the global distribution of SARS-CoV-2 infections in animals" (https://figshare.com/s/8df40ccaa4fd00780650). This repository contains the complete bibliography of 239 scientific papers and 15 World Organisation for Animal Health (WOAH) reports used to compile these infection records, along with the methodological details of data extraction and validation procedures.

**Table S2:** Distribution of high-risk areas in habitats of susceptible animals, categorized by country.

| **Country** | **Total patches** | **High risk cell number**  **in BRT** | **High risk cell number in XGBoost** | **High risk cell number**  **in RF** | **Percentage of high risk cell number**  **(BRT,XGBoost and RF)** |
| --- | --- | --- | --- | --- | --- |
| United States | 16561 | 289 | 1534 | 1120 | 1.75%, 9.26%, 6.76% |
| India | 4132 | 254 | 305 | 247 | 6.15%, 7.38%, 5.98% |
| China | 15323 | 174 | 204 | 139 | 1.34%, 1.33%, 0.91% |
| Brazil | 11023 | 133 | 63 | 97 | 1.21%, 0.57%, 0.88% |
| Bangladesh | 204 | 88 | 59 | 50 | 43.14%, 28.92%, 24.51% |
| Germany | 683 | 59 | 286 | 250 | 8.64%, 41.87%, 36.60% |
| United Kingdom | 338 | 43 | 79 | 96 | 12.72%, 23.37%, 28.40% |
| Indonesia | 1716 | 41 | 38 | 45 | 2.39%, 2.21%, 2.62% |
| Vietnam | 359 | 40 | 43 | 34 | 11.14%, 11.98%, 9.47% |
| Belgium | 83 | 33 | 51 | 53 | 39.76%, 61.45%, 63.86% |
| Italy | 373 | 33 | 56 | 55 | 8.85%, 15.01%, 14.75% |
| Spain | 754 | 18 | 101 | 41 | 2.39%, 13.40%, 5.44% |
| Lithuania | 154 | 5 | 75 | 38 | 3.25%, 48.70%, 24.68% |
| Poland | 632 | 14 | 46 | 58 | 2.22%, 7.28%, 9.18% |
| Mexico | 2578 | 26 | 43 | 31 | 1.01%, 1.67%, 1.20% |
| France | 916 | 17 | 54 | 79 | 1.86%, 5.90%, 8.62% |
| Switzerland | 104 | 23 | 52 | 52 | 22.12%, 50.00%, 50.00% |

**Table S3:** Categorization of areas by country based on projected levels of risk.

| **Country** | **Total cell number** | **High risk**  **percentage** | **High risk cell number** | **Moderate risk cell number** | **Minimal risk cell number** | **Provisionally free cell number** | **Free cell number** |
| --- | --- | --- | --- | --- | --- | --- | --- |
| United States | 16561 | 3.85% | 637 | 1594 | 1340 | 1773 | 11207 |
| India | 4132 | 5.88% | 243 | 949 | 699 | 1339 | 902 |
| China | 15323 | 1.01% | 154 | 1032 | 403 | 2137 | 11597 |
| Germany | 683 | 21.82% | 149 | 137 | 275 | 100 | 22 |
| Brazil | 11023 | 0.76% | 84 | 439 | 211 | 1333 | 8956 |
| United Kingdom | 337 | 18.99% | 64 | 85 | 53 | 92 | 43 |
| Bangladesh | 204 | 29.90% | 61 | 28 | 86 | 5 | 24 |
| Belgium | 83 | 55.42% | 46 | 17 | 19 | 1 | 0 |
| Italy | 373 | 12.33% | 46 | 146 | 69 | 88 | 24 |
| Switzerland | 104 | 43.27% | 45 | 11 | 20 | 13 | 15 |
| France | 916 | 4.37% | 40 | 324 | 78 | 415 | 59 |
| Indonesia | 1716 | 2.33% | 40 | 50 | 52 | 84 | 1490 |
| Vietnam | 359 | 11.14% | 40 | 36 | 24 | 74 | 185 |
| Poland | 632 | 5.38% | 34 | 309 | 94 | 185 | 10 |
| Mexico | 1578 | 1.20% | 31 | 140 | 75 | 277 | 2055 |
| Spain | 754 | 4.11% | 31 | 293 | 125 | 253 | 52 |
| Japan | 377 | 7.96% | 30 | 48 | 22 | 132 | 145 |
| Canada | 23445 | 1.15% | 27 | 128 | 43 | 289 | 22958 |
| Pakistan | 1281 | 2.11% | 27 | 121 | 57 | 160 | 916 |
| Netherlands | 47 | 55.32% | 26 | 10 | 9 | 2 | 0 |

**Table S4:** Comparison of the capabilities of 27 variables as drivers of SARS-CoV-2 infection in animals across three models.

| Driver types | Variables and units | BRT | | | XGBoost | | | RF | | |
| --- | --- | --- | --- | --- | --- | --- | --- | --- | --- | --- |
|  |  | mean | max | min | mean | max | min | mean | max | min |
| Accessibility | Accessibility (h) | 0.33 | 0.51 | 0.16 | 0.11 | 0.14 | 0.08 | 0.53 | 0.67 | 0.25 |
| Population density | Population density (km^-2^) | 0.32 | 0.50 | 0.15 | 0.08 | 0.11 | 0.05 | 0.14 | 0.42 | 0.04 |
| COVID-19 mortality | Mortality (%) | 0.04 | 0.07 | 0.02 | 0.06 | 0.08 | 0.03 | 0.03 | 0.05 | 0.01 |
| Livestock density | Horses (km^-2^) | 0.02 | 0.04 | 0.00 | 0.05 | 0.07 | 0.03 | 0.02 | 0.04 | 0.01 |
|  | Ducks (km^-2^) | 0.03 | 0.05 | 0.01 | 0.04 | 0.07 | 0.03 | 0.03 | 0.05 | 0.01 |
|  | Goats (km^-2^) | 0.01 | 0.02 | 0.00 | 0.03 | 0.05 | 0.01 | 0.03 | 0.05 | 0.01 |
|  | Pigs (km^-2^) | 0.03 | 0.06 | 0.01 | 0.04 | 0.07 | 0.02 | 0.02 | 0.03 | 0.01 |
|  | Chickens (km^-2^) | 0.02 | 0.03 | 0.01 | 0.03 | 0.05 | 0.02 | 0.01 | 0.02 | 0.01 |
|  | Sheep (km^-2^) | 0.02 | 0.03 | 0.01 | 0.04 | 0.06 | 0.02 | 0.02 | 0.03 | 0.01 |
|  | Cattle (km^-2^) | 0.01 | 0.02 | 0.00 | 0.04 | 0.07 | 0.03 | 0.01 | 0.02 | 0.01 |
|  | Buffaloes (km^-2^) | 0.00 | 0.02 | 0.00 | 0.02 | 0.05 | 0.01 | 0.01 | 0.01 | 0.00 |
| Landscape characteristics | PD (100hm^-1^)[1] | 0.02 | 0.04 | 0.01 | 0.03 | 0.04 | 0.01 | 0.01 | 0.03 | 0.01 |
|  | LSI[2] | 0.01 | 0.02 | 0.00 | 0.02 | 0.04 | 0.00 | 0.01 | 0.03 | 0.01 |
|  | MPFD[3] | 0.01 | 0.03 | 0.00 | 0.03 | 0.04 | 0.00 | 0.02 | 0.03 | 0.01 |
|  | SHDI[4] | 0.00 | 0.01 | 0.00 | 0.01 | 0.02 | 0.00 | 0.01 | 0.01 | 0.00 |
|  | ED (m*hm^-1^)[5] | 0.01 | 0.03 | 0.01 | 0.03 | 0.04 | 0.02 | 0.01 | 0.02 | 0.01 |
|  | LPI (%)[6] | 0.01 | 0.03 | 0.00 | 0.03 | 0.04 | 0.01 | 0.01 | 0.01 | 0.01 |
|  | NP[7] | 0.01 | 0.02 | 0.00 | 0.02 | 0.03 | 0.01 | 0.01 | 0.02 | 0.00 |
| Susceptible animals distribution | Animal type | 0.00 | 0.01 | 0.00 | 0.01 | 0.02 | 0.00 | 0.01 | 0.01 | 0.00 |
| Climatic condition | Precipitation (mm) | 0.02 | 0.05 | 0.00 | 0.05 | 0.07 | 0.03 | 0.02 | 0.03 | 0.01 |
|  | Maximum temperature (℃) | 0.01 | 0.04 | 0.00 | 0.04 | 0.05 | 0.02 | 0.01 | 0.02 | 0.01 |
|  | Solar radiation (MJ/m^2^) | 0.02 | 0.04 | 0.01 | 0.04 | 0.06 | 0.02 | 0.00 | 0.03 | 0.01 |
|  | Water vapor pressure(hPa) | 0.01 | 0.03 | 0.00 | 0.04 | 0.07 | 0.02 | 0.01 | 0.02 | 0.01 |
|  | Minimum temperature (℃) | 0.01 | 0.04 | 0.00 | 0.02 | 0.04 | 0.01 | 0.01 | 0.02 | 0.00 |
|  | Average temperature (℃) | 0.01 | 0.04 | 0.00 | 0.02 | 0.04 | 0.00 | 0.01 | 0.02 | 0.00 |
|  | wind speed (m*s^-1^) | 0.01 | 0.03 | 0.00 | 0.05 | 0.07 | 0.03 | 0.00 | 0.02 | 0.01 |
| Biodiversity | Biodiversity intactness index | 0.02 | 0.04 | 0.00 | 0.03 | 0.05 | 0.02 | 0.01 | 0.02 | 0.01 |

Landscape characteristic abbreviations:

[1]PD = Patch Density (number of patches per 100 hectares);

[2]LSI = Landscape Shape Index (measure of landscape shape complexity);

[3]MPFD = Mean Patch Fractal Dimension (measure of patch shape complexity);

[4]SHDI = Shannon's Diversity Index (measure of landscape diversity);

[5]ED = Edge Density (total length of edge per hectare);

[6]LPI = Largest Patch Index (percentage of landscape occupied by the largest patch);

[7]NP = Number of Patches (total number of patches in the landscape).

These metrics were calculated using a 100m resolution raster digital map from the Copernicus Global Land Service to characterize landscape heterogeneity and fragmentation patterns.

**Table S5:** Categorization of susceptible species distribution by country at different risk levels.

| **Animals** | **Total habitat cell number** | **High risk countries** | **High risk**  **cell number** | **Moderate risk cell number** | **Minimal risk cell number** | **Provisionally free cell number** | **Free**  **cell number** |
| --- | --- | --- | --- | --- | --- | --- | --- |
| American mink  (*Neovison vison*) | 30320 | Canada  United States | 613 | 1573 | 1309 | 1804 | 25021 |
| Black-footed Ferret  (*Mustela nigripes*) | 4406 | Canada  Mexico  United States | 43 | 154 | 73 | 340 | 3794 |
| Western Gorilla  (*Gorilla gorilla*) | 972 | Cameroon | 0 | 0 | 2 | 2 | 968 |
| Lion  (*Panthera leoPanthera leo*) | 6559 | South Africa | 0 | 14 | 4 | 86 | 6455 |
| Snow Leopard  (*Panthera uncia*) | 7633 | Kazakhstan | 1 | 30 | 6 | 110 | 7486 |
| Tiger  (*Panthera tigris*) | 3231 | Bangladesh  India  Malaysia  Nepal | 18 | 95 | 237 | 497 | 2384 |
| White-tailed Deer  (*Odocoileus virginianus*) | 23920 | Brazil  Canada  Colombia  Guatemala  Mexico  United States | 674 | 1869 | 1461 | 2409 | 17507 |
| Asian Small-clawed Otter  (*Aonyx cinereus*) | 6289 | Bangladesh  China  India  Indonesia  Malaysia  Nepal  Thailand  Vietnam | 223 | 452 | 434 | 1105 | 4075 |
| Puma  (*Puma concolor*) | 32198 | Argentina  Brazil  Chile  Colombia  Ecuador  Guatemala  Mexico  United States | 188 | 969 | 460 | 2744 | 27837 |

**Table S6:** Distribution of high-risk areas in habitats of susceptible animals, categorized by country.

| **Animals** | **Total habitat patches** | **High risk cell number**  **in BRT** | **High risk cell number in XGBoost** | **High risk cell number**  **in RF** |
| --- | --- | --- | --- | --- |
| American mink(*Neovison vison*) | 30320 | Canada(20)  United States(252) | Canada(38)  United States(1439) | Canada(37)  United States(1046) |
| Black-footed Ferret(*Mustela nigripes*) | 4406 | Canada(2)  Mexico(2)  United States(30) | Canada(2)  Mexico(2)  United States(69) | Canada(3)  Mexico(2)  United States(50) |
| Western Gorilla(*Gorilla gorilla*) | 972 | Cameroon(1) | 0 | 0 |
| Lion(*Panthera leoPanthera leo*) | 6559 | Kenya(1) | 0 | South Africa(1) |
| Snow Leopard(*Panthera uncia*) | 7633 | Kazakhstan(1) | Kazakhstan(1) | Kazakhstan(1) |
| Tiger(*Panthera tigris*) | 3231 | Bangladesh(1)  India(23)  Malaysia(1)  Nepal(2) | Bangladesh(1)  India(19)  Malaysia(1)  Nepal(2) | Bangladesh(1)  India(13)  Malaysia(1) |
| White-tailed Deer(*Odocoileus virginianus*) | 23930 | Brazil(1)  Canada(20)  Colombia(11)  Costa Rica(1)  EI Salvador(1)  Guatemala(2)  Mexico(26)  United States(271)  Venezuela(1) | Canada(38)  Colombia(6)  Costa Rica(1)  Guatemala(2)  Mexico(42)  United States(1485)  Venezuela(1) | Canada(37)  Colombia(6)  Costa Rica(1)  EI Salvador(1)  Guatemala(2)  Mexico(29)  United States(1073)  Venezuela(2) |
| Asian Small-clawed Otter(*Aonyx cinereus*) | 6289 | Bangladesh (50)  Cambodia(1)  China(31)  India(112)  Indonesia(6)  Malaysia(3)  Nepal(8)  Thailand(8)  Vietnam(40) | Bangladesh(37)  Cambodia(1)  China(32)  India(128)  Indonesia(5)  Malaysia(3)  Nepal(9)  Thailand(9)  Vietnam(43) | Bangladesh(32)  Cambodia(1)  China(22)  India(91)  Indonesia(5)  Malaysia(3)  Nepal(3)  Thailand(7)  Vietnam(34) |
| Puma(*Puma concolor*) | 32198 | Argentina(5)  Bolivia(1)  Brazil(133)  Chile(4)  Colombia(11)  Costa Rica(1)  Ecuador(2)  EI Salvador(1)  Guatemala(2)  Mexico(17)  Paraguay(2)  United States(34)  Venezuela(1) | Argentina(5)  Bolivia(1)  Brazil(63)  Chile(3)  Colombia(6)  Costa Rica(1)  Ecuador(2)  Guatemala(2)  Mexico(31)  Paraguay(1)  United States(91)  Venezuela(1) | Argentina(4)  Bolivia(1)  Brazil(97)  Chile(5)  Colombia(6)  Costa Rica(1)  Ecuador(3)  EI Salvador(1)  Guatemala(2)  Mexico(22)  Paraguay(2)  United States(88)  Venezuela(2) |
